# Connections Across Open Water: A Bi‐Organelle, Genomics‐Scale Assessment of Atlantic‐Wide Population Dynamics in a Pelagic, Endangered Apex Predator Shark (*Isurus oxyrinchus*)

**DOI:** 10.1111/eva.70071

**Published:** 2025-01-22

**Authors:** Andrea M. Bernard, Marissa R. Mehlrose, Kimberly A. Finnegan, Bradley M. Wetherbee, Mahmood S. Shivji

**Affiliations:** ^1^ Save Our Seas Foundation Shark Research Center, Halmos College of Arts & Sciences Nova Southeastern University Dania Florida USA; ^2^ Guy Harvey Research Institute, Halmos College of Arts & Sciences, Nova Southeastern University Dania Florida USA; ^3^ Department of Biological Sciences University of Rhode Island Kingston Rhode Island USA

**Keywords:** chromosomal structural variant, management, mitochondrial DNA, population genetic structure, shortfin mako shark, single nucleotide polymorphism

## Abstract

Large‐bodied pelagic sharks are key regulators of oceanic ecosystem stability, but highly impacted by severe overfishing. One such species, the shortfin mako shark ( *Isurus oxyrinchus* ), a globally widespread, highly migratory predator, has undergone dramatic population reductions and is now Endangered (IUCN Red List), with Atlantic Ocean mako sharks in particular assessed by fishery managers as overfished and in need of urgent, improved management attention. Genomic‐scale population assessments for this apex predator species have not been previously available to inform management planning; thus, we investigated the population genetics of mako sharks across the Atlantic using a bi‐organelle genomics approach. Complete mitochondrial genome (mitogenome) sequences and genome‐wide SNPs from sharks distributed across the Atlantic revealed contrasting patterns of population structure across marker types. Consistent with this species' long‐distance migratory capabilities, SNPs showed high connectivity and Atlantic panmixia overall. In contrast, there was matrilineal population genetic structure across Northern and Southern Hemispheres, suggesting at least large regional‐scale female philopatry. Linkage disequilibrium network analysis indicated that makos possess a chromosomal inversion that occurs Atlantic wide, a genome feature that may be informative for evolutionary investigations concerning adaptations and the global history of this iconic species. Mitogenome diversity in Atlantic makos was high compared to other elasmobranchs assessed at the mitogenome level, and nuclear diversity was high compared to the two other, highly migratory pelagic shark species assessed with SNPs. These results support management efforts for shortfin makos on at least Northern versus Southern Hemisphere scales to preserve their matrilineal genetic distinctiveness. The overall comparative genetic diversity findings provide a baseline for future comparative assessments and monitoring of genetic diversity, as called for by the United Nations Convention on Biological Diversity, and cautious optimism regarding the health and recovery potential of Atlantic shortfin makos if further population declines can be halted.

## 1 Introduction

During the past five decades, pelagic elasmobranchs (sharks and rays) have undergone alarming global declines (~70%), with three‐quarters of these oceanic, wide‐ranging, and mostly highly mobile species now at risk of extinction (Pacoureau et al. 2021; Juan‐Jordá et al. 2022). This dramatic and ongoing loss in abundance is mainly driven by pervasive overfishing and bycatch (Dulvy et al. 2008; Pacoureau et al. 2021). These fisheries interactions with pelagic elasmobranchs typically occur on the high seas beyond national jurisdictions (Cronin et al. 2023), making coordinated regional fisheries management and conservation efforts essential to stem continued losses of these ecologically important species and allow their recovery. Sustainable conservation management is aided by an understanding of the biology of these species, including foundational information about their genetic spatial connectivity and diversity.

With few strong barriers to dispersal in open oceans, the a priori expectation for large‐bodied pelagic sharks, which often possess long‐distance dispersal propensities as adults and ocean basin or worldwide distributions, is that low levels of population genetic differentiation exist across their range. However, nuclear and/or mitochondrial DNA population genetic structure is increasingly being identified in these highly migratory species, e.g., white sharks ( *Carcharodon carcharias* ; Blower et al. 2012; Bernard et al. 2018), night sharks ( *Carcharhinus signatus* ; Domingues et al. 2019), silky sharks ( *Carcharhinus falciformis* ; Clarke et al. 2015; Kraft et al. 2020), blue sharks ( *Prionace glauca* ; Nikolic et al. 2023), pelagic thresher sharks ( *Alopias pelagicus* ; Cardeñosa, Hyde, and Caballero 2014), oceanic whitetip sharks ( *Carcharhinus longimanus* ; Camargo et al. 2016; Ruck et al. 2024), tiger sharks ( *Galeocerdo cuvier* ; Bernard et al. 2016, 2021; Holmes et al. 2017; Sort et al. 2021), porbeagle sharks ( *Lamna nasus* ; González et al. 2021), and shortfin mako sharks ( *Isurus oxyrinchus* ; Corrigan et al. 2018; González et al. 2023; Vella and Vella 2023). It is anticipated that investigations employing newer generation, whole genome‐based, high‐resolution markers (e.g., single nucleotide polymorphisms (SNPs) and/or whole mitogenomes) will further reveal finer geographic‐scale population differentiation undetected in these species previously with traditional genetic approaches (e.g., Kraft et al. 2020; Bernard et al. 2021; Green et al. 2022; Nikolic et al. 2023). Use of such genome‐level markers will also provide greater precision for elucidating the population histories of species (e.g., Thrasher et al. 2018; Bohling et al. 2019; Zimmerman, Aldridge, and Oyler‐McCance 2020) and improve investigations of broad evolutionary questions concerning adaptive genetic differences (e.g., Vendrami et al. 2019; Junge et al. 2019; Zimmerman, Aldridge, and Oyler‐McCance 2020) and the chromosomal architecture and genomic complexity of species (Kemppainen et al. 2015; Wellenreuther et al. 2019). Furthermore, robust understanding of matrilineal population genetic dynamics will benefit from whole mitochondrial genome sequence‐based approaches, given that past assessments have demonstrated that population and genetic diversity inferences can vary depending on the mitochondrial gene/region analyzed. This variability can result from the reduced information and associated limited statistical power obtained when using short mitochondrial sequences, and varying mutation rates and evolutionary selection in individual mitochondrial regions (Dudgeon, Broderick, and Ovenden 2009; Teacher et al. 2012; Feutry et al. 2014; Lou et al. 2018; Johri et al. 2019; Kraft et al. 2020).

With concerns mounting about the high extinction risks for pelagic sharks, there is a need to apply genomic approaches to better reveal the population dynamics of these species for conservation management and improve understanding of their general evolutionary history. A pelagic species of high conservation concern is the shortfin mako shark ( *Isurus oxyrinchus* ), a large‐bodied, highly migratory, apex predator that is targeted for its meat and fins in fisheries throughout its global distribution. Shortfin makos (hereafter, makos) are classified as “Endangered” on the International Union for Conservation of Nature's (IUCN) Red List of Threatened Species (Rigby et al. 2019) and listed on Appendix II of the Convention on International Trade in Endangered Species (CITES) due to population declines attributed to widespread overfishing. Within Atlantic waters, the mako's population status is particularly dire (Sims, Mucientes, and Queiroz 2018, 2021). The most recent stock assessment indicates Northern Atlantic mako sharks are overfished, and there is high uncertainty about the status of the South Atlantic stock (ICCAT 2017). Furthermore, independent, satellite tagging‐derived data for makos in the North Atlantic indicate that fishing mortality rates may be up to ten times higher than previously estimated by pre‐2017 Atlantic stock assessment models (Byrne et al. 2017).

Conventional tagging‐ and satellite telemetry‐based studies of makos in the Atlantic indicate complex dispersal dynamics with individuals showing a variety of dispersal behaviors, including long‐term area residency, region‐specific movement patterns, and long‐distance, seasonal return migrations (Vaudo et al. 2017; Byrne et al. 2019; Kohler and Turner 2019; Santos et al. 2021; Gibson et al. 2021). Notably, all the combined tagging data have revealed only a single case of trans‐equatorial, North–South hemisphere movements (Santos et al. 2021), leading to a prediction of at least inter‐hemispheric, population genetic structure in the Atlantic.

The only genomic‐level assessments reported for the mako shark thus far are a chromosomal‐level genome assembly (Stanhope et al. 2023) and the development of a panel of SNP markers (Domingues et al. 2022), but neither of these studies conducted population‐level assessments. All genetic assessments of mako population dynamics in various parts of the species' global distribution have thus far relied on sections of mitochondrial DNA sequences from the control region and/or some protein‐coding genes (Heist, Musick, and Graves 1996; Taguchi, Kitamura, and Yokawa 2011; Corrigan et al. 2018; González et al. 2023; Vella and Vella 2023), and/or a modest number of nuclear microsatellite markers (Schrey and Heist 2003; Corrigan et al. 2018). Microsatellite DNA assessments suggest that makos comprise a globally panmictic population (Schrey and Heist 2003; Corrigan et al. 2018), though these results are tempered by the use of either a few microsatellite markers (i.e., Schrey and Heist 2003—four microsatellites) or limited spatial distribution of Atlantic sharks in the assessments (Corrigan et al. 2018—focused on Indo‐Pacific‐wide makos, with Atlantic samples only from the NE Atlantic). Consistent with predictions from Atlantic mako tagging‐based findings, early mitochondrial restriction fragment polymorphism (RFLP) work (Heist, Musick, and Graves 1996) suggested North and South Atlantic makos to be genetically differentiated. Recent work using short sequences of the mitochondrial control region (CR) and/or Cytochrome Oxidase I gene with sampling only from the eastern Atlantic and/or Mediterranean (González et al. 2023) supports mako northern vs. southern hemisphere matrilineal structure in the eastern Atlantic.

The imperiled status of the mako, its global distribution, and long‐distance migrations necessitate a cooperative international approach to its recovery and sustainable management. Improved management efforts and assessment of adaptive evolutionary potential underscore the need for a high‐resolution genetic survey of the population dynamics of this species. Herein, we report an assessment of the population genetics of the mako shark, as derived from single nucleotide polymorphisms and complete mitochondrial genome sequences. Here, we focus on an Atlantic‐wide assessment, including population genomic structure, sex‐biased dispersal, comparative genetic diversity, and genomic architecture of this iconic, endangered, apex predator.

## 2 Materials and Methods

### 2.1 Shortfin Mako Atlantic Distribution, Sample Collection, and Genomic DNA Extraction

**FIGURE 1:**
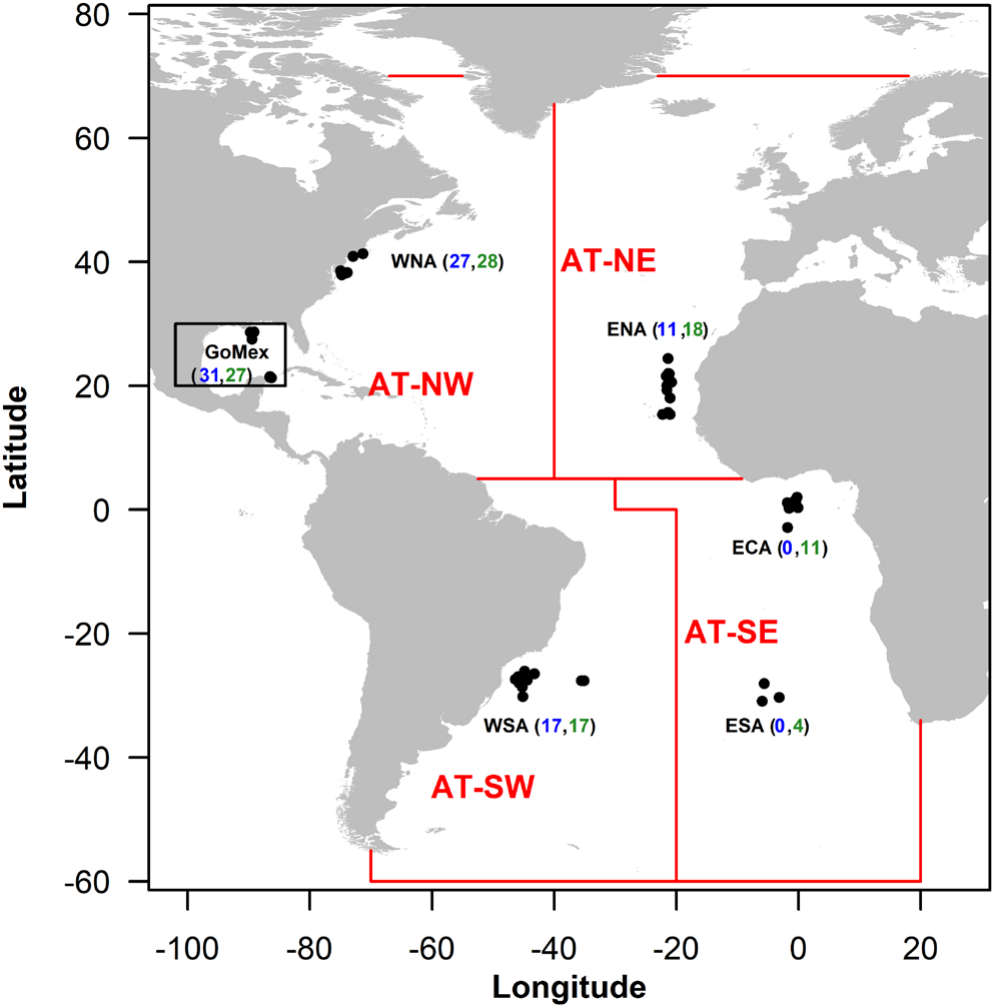
Map of shortfin mako shark ( *Isurus oxyrinchus* ) a priori defined, Atlantic sampling groupings (hereafter referred to as “subpopulations”): WNA, western North Atlantic; GoMex, Gulf of Mexico and Isla Mujeres, Mexico; WSA, western South Atlantic; ENA, eastern North Atlantic; ECA, eastern Central Atlantic; ESA, eastern South Atlantic. Individual sample locations (i.e., exact capture location or beginning of fishing transect) are represented by a black dot, and a black rectangular box represents a sample group where only general capture location is available. Numbers in brackets represent final shark sample sizes used in downstream analysis (Blue: Complete mitochondrial genomes; Green: Post‐filtered sample sizes for single nucleotide polymorphisms (SNPs) dataset). Red lines delimit ICCAT stock/statistical management areas for sharks; red text indicate abbreviations of ICCAT stock/statistical management areas: AT‐NE, Atlantic Northeast; AT‐NW, Atlantic Northwest; AT‐SW, Atlantic Southwest; AT‐SE, Atlantic Southeast.

The mako's Atlantic distribution spans 60°N to 50°S in oceanic and shelf waters (Ebert, Dando, and Fowler 2021). Tissue samples were obtained from 144 sharks across most of this latitudinal range, captured between 2004 and 2018 from six general Atlantic locations (hereafter six “subpopulations”). Individual shark sample subpopulation locations and final sample sizes of SNP genotyped and whole mitogenome sequenced sharks are shown in Figure 1. Sample metadata (shark sex, size, sequence reads, GenBank Accession numbers) are shown in Appendix S1: Table S1. Samples were typically obtained from fisheries or research telemetry tagging expeditions (fin clips or muscle tissue plugs), and preserved in 99% undenatured ethanol. Genomic DNA was extracted from tissues using either DNeasy Blood and Tissue Kits according to manufacturer's instructions (QIAGEN Inc.) or by the lysis extraction method of Wilson, Lavender, and Black (2007) as modified by Bernard et al. (2022). Following tissue lysis, 4‐μL of QIAGEN RNAse A was added to the lysate of all samples used for downstream SNP library preparation. A Qubit 3 Fluorometer (Invitrogen) was used to quantify all genomic DNA extracts.

### 2.2 Mitogenome DNA: Long‐PCR, Library Preparation, and Sequencing

Complete mako mitochondrial genomes (mitogenomes) were amplified via long‐polymerase chain reaction (long‐PCR) in four to eight individual fragments of varying sequence length (1323–5852 bp) (details described in Appendix S1: Methods S1, Table S2). Mitochondrial DNA library preparation for sequencing was performed using the Nextera XT DNA Library Preparation Kit (Illumina Inc.) following the manufacturer's guidelines, and included a dilution of each amplified fragment to a concentration of 0.2 ng/μL and the pooling of all amplicons belonging to a single sample (individual shark). Final DNA library size was assessed on an Agilent TapeStation (Agilent Technologies Inc.) followed by paired‐end (2 × 150 PE) sequencing on an Illumina MiSeq.

### 2.3 Mitogenome DNA: Sequence Read Quality Filtering, Assembly, and Population Genetic Analysis

Within Geneious Prime 2021.2.2 (Kearse et al. 2012), raw Illumina PE reads were merged and then trimmed using BBDuk 38.84 (Bushnell, Rood, and Singer 2017). Adapter sequences (All Truseq, Nextera, and PhiX adapters) and low‐quality nucleotides (minimum Phred quality score of 30) were trimmed and reads spanning less than 75 bp were discarded. Mitogenome sequences were assembled using the “Map to Reference” feature in Geneious (reference sequence GB# KF361861; Chang et al. 2015) and the Geneious Mapper feature. Consensus sequences were generated from assembled contigs assuming a minimum of 10× coverage (settings: Threshold = Highest Quality (60%); Assign Quality = Total). Where 10× coverage across the mitogenome was not achieved (*n* = 6 sharks), mitochondrial DNA long‐PCR amplification, library preparation, and sequencing were repeated, with Illumina reads generated across different MiSeq runs combined to achieve higher sequence coverage (*n* = 4 sharks), or gaps in sequence were filled via the process of Sanger Sequence primer walking (*n* = 2 sharks; see Appendix S1: Methods S2, Tables S1 and S2). The above methods resolved 85 complete assembled mitogenomes from four of the six subpopulations (see Section 3). Resultant assembled mitogenome sequences were annotated using Mitoannotator 3.72 (Iwasaki et al. 2013) and aligned in Geneious using the Muscle 3.8.425 (Edgar 2004) feature. Input files for downstream analyses were generated using FaBox 1.61 (Villesen 2007).

Overall, hierarchical (see groups below) and per collection site genetic diversity estimates (number of haplotypes, haplotype, and nucleotide diversity, and number of polymorphic sites), nucleotide composition, and demographic parameters (Tajima's *D*, Tajima 1989; Fu's *F*_S_, Fu 1997) were determined for complete mitogenome sequences using the program Arlequin 3.5.2.1 (Excoffier and Lischer 2010); nucleotide ambiguities (degenerate bases) were coded as missing data and significance of demographic parameters was estimated by 10,000 simulated samples. Two hierarchical Analysis of Molecular Variance (AMOVAs) were performed using Arlequin with the four collection sites grouped according to: (1) current International Commission for the Conservation of Atlantic Tunas (ICCAT) management boundaries for Atlantic makos (see Figure 1; Northern Atlantic vs. Southern Atlantic with 5°N as the latitudinal stock limits), and (2) *potential* Western vs. Eastern Atlantic mako groupings using ICCAT's stock/statistical longitudinal boundaries for sharks as our a priori longitudinal division of sharks (see Figure 1 for locations of ICCAT stock/statistical boundaries). AMOVAs were performed assuming pairwise genetic distance (ϕ_ST_), nucleotide ambiguities coded as missing data, and significance was determined using 10,000 permutations. Likewise, Arlequin was used to estimate pairwise values of genetic differentiation (pairwise genetic distance: ϕ_ST_; ambiguities coded as missing data) between mako hierarchical groups and the four subpopulations. Significance was determined using 10,000 permutations and these values were adjusted using Benjamini and Hochberg's (1995) false discovery rate (FDR) correction as implemented in R version 4.0.5 (R Core Team 2021; function: “p.adjust”). Phylogeographic relationships among complete mitogenome haplotypes were visualized by means of a Median‐Joining Network (Bandelt, Forster, and Röhl 1999) using the program PopART 1.7 (Leigh and Bryant 2015); all other publicly available globally sourced mako whole mitogenome sequences were included in the network to orient our sequences in a global phylogeographic context [Chang et al. 2015—Pacific: KF361861; Gorman et al. 2019—WNA: MF537044; Mehlrose et al. 2022—WNA: MZ923833, ENA: MZ923832, and western South Pacific (New Zealand): MZ923831].

### 2.4 Double‐Digest Restriction Site‐Associated DNA Sequencing (RADseq): Library Preparation, Sequencing, and De Novo SNP Assembly

SNP libraries were generated for 135 mako samples (plus nine technical replicates, total *n* = 144) according to Peterson et al.'s (2012) double‐digest RADseq protocol, using aliquots of 200 ng of template genomic DNA, and the restriction enzymes SphI (Life Technologies Inc.) and MluC1 (New England Biolabs Inc.). Following digestion, samples were purified using AMPure XP beads (Beckman Coulter Inc.) and individual barcode (P1; Sigma‐Aldrich Inc.) and universal adapters (P2; Sigma‐Aldrich Inc.) were ligated to enzyme cut sites (SphI and MluC1 restriction enzymes, respectively). Eight unique P1 adapters were adopted herein; thus, each final indexed library contained eight individual sharks (each with a variable P1 adapter). Size selection of RAD libraries (495–605 bp in length) was performed using a Pippin Prep DNA Size Selection System (Sage Science Inc.) and PE sequencing (2 × 150 PE) was performed by GENEWIZ (Azenta US Inc.) on three lanes of an Illumina HiSeq 4000. Quality of raw Illumina reads was assessed using FastQC 0.11.8 (Andrews 2010), followed by de novo SNP discovery using Stacks 2.54 (Catchen et al. 2013; Rochette, Rivera‐Colón, and Catchen 2019) as outlined in Appendix S1: Methods S3.

### 2.5 RADseq: SNP Bioinformatics Quality Filtering, Relatedness, and Linkage Disequilibrium Network Analysis

Secondary filtering of the SNP dataset using the software vcftools 0.1.13 (Danecek et al. 2011) was performed in a series of successive (and sometimes repeated) steps to include only: (1) loci with a minimum of 8× coverage, (2) loci genotyped in at least 70% of individuals and possessing a minor allele frequency of 5%, (3) individuals genotyped across at least 70% of SNP loci, (4) SNP loci with a mean depth of at least 10X coverage across individuals, (5) loci genotyped in at least 70% of individuals and possessing a minor allele frequency of 5%, and (6) SNP loci whose maximum mean depth of coverage was no more than two times the value of the mean coverage across loci.

To test for duplicate samples (i.e., the same shark sampled twice in the field), and to evaluate the genotyping similarity of RAD library technical sample replicates, the R package strataG 2.0.2 (Archer, Adams, and Schneiders 2017) was employed; where duplicate genotypes were identified (shared genotype > 80%), the individual shark with the highest rate of missing data was discarded. Input files for downstream analysis were generated using the R package vcfR 1.12.0 (Knaus and Grünwald 2017). Relatedness between samples was assessed using the R package SNPRelate 1.24.0 (Zheng et al. 2012) and via implementing the KING identity‐by‐descent (IBD) method.

To test for adaptive selection and/or chromosomal structural variants (i.e., the non‐random association of alleles within the RAD dataset), we performed linkage disequilibrium network analysis (LDna) using the methodology of Kemppainen et al. (2015) and the R package LDna 0.64. The LDna method relies on network analyses to identify clusters of loci in high linkage disequilibrium (LD) and exploits the assumption that any LD‐locus “outlier” clusters that remain highly distinct across a vast range of LD values likely represent groups of loci that exhibit and share a unique population genetic signal (Kemppainen 2014). Thus, the purpose of the LDna method is to identify and extract these tightly‐linked [i.e., single outlier clusters (SOC)] sets of outlier loci so that they can be explored further via downstream population genetic analyses (Kemppainen 2014). To perform LDna on our dataset, we generated a matrix of pairwise linkage disequilibrium (LD; *r* ^2^) values between all SNP loci using the R package pegas 1.0‐1 (Paradis 2010). LDna was run using this LD matrix as input and assuming a minimum edge number of 52 [per Kemppainen et al. 2015 ~1% of dataset] and a phi value of 8. SNP loci appearing in the identified SOC (see Section 3) were extracted and analyzed via a principal component analysis (PCA) using the R package adegenet 2.1.3 (function: “dudi.pca”; Jombart 2008; Jombart and Ahmed 2011). For each of the three SOC PCA‐resolved clusters (see Section 3), estimates of genetic diversity [observed (*H*_O_) and expected heterozygosity (*H*_E_) (adegenet) and the inbreeding coefficient (*F*_IS_; hierfstat 0.5–10; Goudet 2004)] were obtained. Consensus sequences of all SOC RAD loci were: (1) mapped to the mako unannotated National Center for Biotechnology Information (NCBI) scaffold assembly GCA_026770705.1 (5558 individual sequences; Stanhope et al. 2023; NCBI Genome Assembly ASM2677070v1) using Geneious Prime and its “Map to Reference” Geneious Mapper feature. Settings included a minimum mapping quality of 30 and allowed 10% mismatches and 10% gaps per matched read; and (2) compared against a reference chromosomal‐level, annotated (Annotation Release 100) genome assembly of the white shark (GCF_017639515.1) using the NCBI Blastn feature per Domingues et al. (2022). Matches of SOC RAD loci mako consensus sequences to the white shark genome were retained if percent identity was ≥ 95% and the bitscore exceeded 200. Up to three chromosomal matches were retained per consensus sequence. Next, to identify white shark genomic features in close proximity to anchored SOC mako loci, we used the program BEDOPS v. 2.4.39 (Neph et al. 2012) to extract annotations (genomic feature, product, and genetic distance in nucleotides to nearest feature; “closest‐features” command) from the white shark reference genome. Then, as performed in Vaux et al. (2021), for any identified homologous white shark genomic features matching mako consensus sequences, we (where possible) determined the corresponding homologous human NCBI Entrez IDs and searched for these human Entrez IDs in PANTHER 19.0 (Thomas et al. 2022) to identify their potential biological functions (Panther Go‐slim Biological Processes). As stated in Vaux et al. (2021), evolutionarily distant species comparisons (i.e., white shark vs. human) may still be valid if vertebrate gene function is conserved.

To complement the network analysis in the identification of putative outlier loci: (1) BayeScan 2.1 (Foll and Gaggiotti 2008) was run using default settings (prior odds of 100, and an FDR of 5%), and (2) the R package OutFLANK 0.2 (Lotterhos and Whitlock 2015) was used assuming default settings, six a priori subpopulations, and a *q* threshold of 0.05.

### 2.6 SNPs: Population Genetic Structure Analysis

Following LDna and testing for candidate outlier loci, non‐SOC loci (hereafter referred to as the putatively “neutral SNP loci”) were tested for conformation to Hardy–Weinberg equilibrium (HWE) proportions within subpopulations (where sample size was ≥ 15) using the R package pegas (function: “hw.test”); those loci deviating from HWE at *p* < 0.05 after FDR correction within two or more subpopulations were discarded. Estimates of SNP diversity (overall, hierarchical, and per subpopulation), including *H*_O_, *H*_E_, *F*_IS_, allelic richness (*A*_R_; function “allelic. Richness”), and the number of private alleles per subpopulation (*pa*) were estimated using the R packages adegenet (*H*_O_ and *H*_E_), hierfstat (*F*_IS_, *A*_R_), and poppr 2.9.2 (*pa*; Kamvar, Tabima, and Grünwald 2014; Kamvar, Brooks, and Grünwald 2015).

Signals of population genetic structure within neutral SNP loci were assessed using a set of population‐ and individual‐level analyses. Overall and pairwise estimates of genetic differentiation (*F*_ST_; Weir and Cockerham 1984) between subpopulations and hierarchical groupings (as defined above) were generated using the R package strataG and the functions “overallTest” (strataG) and “pairwiseTest” (strataG). Significance was determined by implementing 1000 permutation replicates and pairwise values of significance were adjusted (FDR). Then neutral population genetic structure was further assessed by means of a set of individual‐based cluster analyses. First, a multivariate PCA was performed using the R package adegenet as outlined above. And second, the Maximum Likelihood program Admixture (Alexander, Novembre, and Lange 2009) and its cross‐validation (CV) error approach was adopted to determine the most appropriate number of clusters within the dataset, assuming default settings, and *K* = 1–10.

To test for sex‐biased dispersal in makos, we used the methods outlined in Goudet, Perrin, and Waser (2002) and tested for differences between the two sexes across four metrics: (1) Weir and Cockerham's (1984) *F*_IS_, the inbreeding coefficient, where the dispersing sex should possess a higher value of *F*_IS_ (a heterozygote deficit compared to HWE proportions) compared to the more philopatric sex; (2) the *F*_ST_ statistic, which represents the proportion of the total genetic variance that occurs among populations, where the more philopatric sex should have a larger *F*_ST_ compared to the more dispersing sex; (3) mean corrected Assignment Index (mAIc; Paetkau et al. 1995; Favre et al. 1997), where the assignment probability of the dispersing sex should possess a lower mean AIc than the more philopatric sex; and (4) variance of the corrected Assignment Index (vAIc), where the dispersing sex should have a larger vAIc than the more philopatric sex. The above metrics were estimated using the R package hierfstat (function: “sexbias.test”); statistical significance of estimates between male (*n* = 47) and female (*n* = 58) makos was determined by implementing 1000 permutations.

## 3 Results

### 3.1 Mitogenomes

A total of 85 mako complete mitogenomes were generated, ranging in length from 16,699 to 17,002 bp. Of these mitogenomes, 83 were assembled from Illumina reads, while two were assembled using a combination of Illumina reads and stretches of primer‐walked and assembled Sanger sequences (shark OC‐072, Sanger sequenced 3111‐bp, and shark OC‐365, Sanger sequenced 4146‐bp). In total, 34,329,726 raw Illumina PE reads were recovered across samples, with a mean of 403,879 reads per shark. Following assembly, each mitogenome consisted of an average of 158,171 mapped reads (2 × 150 PE; range: 18,424–993,495 reads; Appendix S1: Table S1). Mean sequence depth across the final, individual mitogenomes ranged from 209× to 11,838×, with a minimum and maximum coverage of 11× and 97,907×, respectively (these coverage statistics do not include the two shark samples requiring Sanger sequences to complete assembly). Along with the 85 complete mitogenome sequences generated herein, all downstream population‐level analyses included the mitogenome sequence of a mako shark (GenBank Accession Number: MZ923832; Mehlrose et al. 2022) from the eastern North Atlantic (ENA) (Figure 1; Appendix S1: Table S1). In the final curated 86 complete mitogenome sequences, evidence of heteroplasmy (i.e., two nucleotides presenting in near equal frequencies at a single position for Illumina‐derived sequences) was found at 21 nucleotide positions across 15 individual sharks: 18 of these ambiguities appeared in protein‐coding genes (ATP6, *n* = 1; COI, *n* = 5; ND1, *n* = 2; ND2, *n* = 1; ND3, *n* = 1; ND4L, *n* = 1; ND4, *n* = 5; ND5, *n* = 2), and the remaining three appeared in the non‐protein‐coding regions t‐RNA‐Phe, 12S rRNA, and the D‐Loop (one in each region). A single ambiguity was found in a Sanger‐sequenced region, presenting as overlapping peaks in the chromatograms. Annotation of the mitogenomes yielded an identical order and complement (13 protein‐coding genes, two ribosomal RNAs, 22 transfer RNAs, and one non‐coding D‐Loop) to other published, globally‐sourced mako mitogenomes (Chang et al. 2015; Gorman et al. 2019; Mehlrose et al. 2022). The overall GC content of mitogenomes was 43.20%.

Atlantic‐wide mitogenome genetic diversity was high. Across the 86 mitogenomes, 76 haplotypes were recovered, separated by 645 polymorphic sites, and consisting of 588 transitions, 58 transversions, and 9 indels. Overall haplotype and nucleotide diversity was 0.997 ± 0.002 and 0.00654 ± 0.00314, respectively (Table 1). Estimates of genetic diversity were similar among the four a priori defined subpopulations and hierarchical groupings (i.e., Northern Atlantic, Southern Atlantic, Eastern Atlantic, and Western Atlantic) from whom mitogenomes were obtained. There was no evidence of population expansion according to the parameters Tajima's *D* and Fu's *F*s, as values did not deviate from neutral expectations (Table 1).

**TABLE 1:** Atlantic shortfin mako shark population genetic diversity statistics by a priori subpopulations and hemispheres as determined from complete mitogenome sequences (*n* = 86) and 4971 genotyped single nucleotide polymorphisms (*n* = 105).

Subpopulation of grouping	Mitogenomes							Single nucleotide polymorphisms (SNPs)				
	*N*	*nh*	*np*	*h*	π	*D* (*p*)	*F*_s_ (*p*)	*n*	*H*_O_	*H*_E_	*A*_R_	*F*_IS_
WNA	27	24	380	0.989 ± 0.015	0.00493 ± 0.00244	−0.635 (0.277)	0.085 (0.498)	28	0.273	0.273	1.295	0.017
GoMex	31	28	435	0.994 ± 0.010	0.00712 ± 0.00349	0.369 (0.708)	0.258 (0.534)	27	0.276	0.276	1.279	0.019
WSA	17	16	406	0.993 ± 0.023	0.00726 ± 0.00367	0.072 (0.584)	0.969 (0.595)	17	0.275	0.271	1.280	0.013
ENA	11	11	310	1.000 ± 0.039	0.00474 ± 0.00250	−1.232 (0.106)	0.054 (0.312)	18	0.283	0.275	1.285	0.008
ECA	—	—	—	—	—	—	—	11	0.270	0.262	1.276	0.019
ESA	—	—	—	—	—	—	—	4	0.299	0.250	1.295	−0.047
Northern Atlantic	69	61	556	0.996 ± 0.003	0.00606 ± 0.00292	−0.433 (0.386)	−4.772 (0.115)	73	0.276	0.279	—	0.018
Southern Atlantic	—	—	—	—	—	—	—	32	0.276	0.276	—	0.018
Western Atlantic	75	66	614	0.996 ± 0.003	0.00672 ± 0.00324	−0.361 (0.426)	−4.653 (0.128)	72	0.275	0.278	—	0.020
Eastern Atlantic	—	—	—	—	—	—	—	33	0.279	0.278	—	0.015
Atlantic Ocean Overall	86	76	645	0.997 ± 0.002	0.00654 ± 0.00314	−0.511 (0.355)	−7.423 (0.066)	105	0.28	0.28	—	0.012

*Note:* Mitogenomes: *A*_R_, mean allelic richness; *D*, Tajima's *D* (Tajima 1989); *p*, *p*‐value; *F*_S_, Fu's *F*_S_ (Fu 1997); *F*_IS_, *H*_O_, observed heterozygosity; *H*_E_, expected heterozygosity; *N*, number of complete mitogenomes; *nh*, number of haplotypes; *np*, number of polymorphic sites; *h*, haplotype diversity; π, nucleotide diversity; SNPs: *n*, number of samples genotyped; inbreeding coefficient. Subpopulation (from Figure 1) and/or Grouping Abbreviations: WNA, western North Atlantic; GoMex, Gulf of Mexico and Isla Mujeres, Mexico; WSA, western South Atlantic; ENA, eastern North Atlantic; *ECA, eastern Central Atlantic; *ESA, eastern South Atlantic; Northern Atlantic (WNA, GoMex, ENA); Southern Atlantic (WSA, *ECA, *ESA); Western Atlantic (WNA, GoMex, WSA); Eastern Atlantic (ENA, *ECA, *ESA). * denote samples from these a priori subpopulations analyzed for SNPs only.

Hierarchical AMOVAs indicated some, albeit limited, evidence of matrilineal population genetic structure. Between‐group variance was maximized (8.43%) when subpopulations were latitudinally partitioned by current ICCAT stock management boundaries (Figure 1) into Atlantic Northern versus Southern Hemisphere groups, although without statistical significance (*p* = 0.252) (Appendix S1: Table S3a). When Atlantic mako subpopulations were grouped into hierarchical groups longitudinally, between‐group variance was negative (−2.34%) and there was no statistically significant genetic differentiation found between Eastern Atlantic versus Western Atlantic subpopulations (*p* = 0.754); however, statistically significant genetic population structure was found *within* these longitudinal hierarchical groups (ϕ‐statistic = 0.084, *p* = 0.009; Appendix S1: Table S3b), indicating the presence of some matrilineal population genetic differentiation among western Atlantic mako subpopulations, since the eastern Atlantic contained only one subpopulation. Pairwise testing for mitogenome genetic differentiation between subpopulations also yielded statistically significant matrilineal differentiation between some Northern versus Southern Atlantic regions (i.e., WNA vs. WSA and ENA vs. WSA; ϕ_ST_ = 0.193 and 0.192, respectively; *p* < 0.05; Table 2). When samples were compared by hemisphere groupings [i.e., Northern (WNA, GoMex, ENA) versus Southern Hemispheres (WSA)], significant genetic differentiation was also found (ϕ_ST_ = 0.108, *p* = 0.006; Table 3). No significant pairwise matrilineal genetic population structure was found between any Western and Eastern Atlantic mako subpopulations (Table 2).

**TABLE 2:** Atlantic shortfin mako shark a priori subpopulation‐level, pairwise values of genetic differentiation (mitogenomes: Φ_ST_; SNPs: *F*_ST_).

Sub‐population comparison	Φ_ST_	*F*_ST_
	Mitogenomes	4971 SNPs
	*n* = 86	*n* = 105
WNA vs. GoMex	**0.059**	0.000
WNA vs. WSA	**0.193** ^a^	0.001
WNA vs. ENA	−0.041	**0.001**
WNA vs. ECA	—	**0.002**
WNA vs. ESA	—	0.001
GoMex vs. WSA	0.015	0.001
GoMex vs. ENA	0.056	**0.001**
GoMex vs. ECA	—	0.001
GoMex vs. ESA	—	0.000
WSA vs. ENA	**0.192**	0.001
WSA vs. ECA	—	0.000
WSA vs. ESA	—	0.000
ENA vs. ECA	—	0.001
ENA vs. ESA	—	−0.001
ECA vs. ESA	—	0.001

*Note:* Values in bold (mitogenome and SNP) indicate non‐adjusted statistical significance at *p* < 0.05.

Abbreviations: ECA, eastern Central Atlantic; ENA, eastern North Atlantic; ESA, eastern South Atlantic; GoMex, Gulf of Mexico and Isla Mujeres, Mexico; WNA, western North Atlantic; WSA, western South Atlantic.

^a^ Indicates mitogenome and SNP statistical significance at *p* < 0.05 after False Discovery Rate correction.

**TABLE 3:** Atlantic shortfin mako shark pairwise values of mitogenomes (Φ_ST_) and nuclear SNPs (*F*_ST_) genetic differentiation among hierarchical groups.

Hierarchical group comparisons	Φ_ST_ (*p*)	*F*_ST_ (*p*)
	Mitogenomes	4971 SNPs
	*n* = 86	*n* = 105
Northern Atlantic vs. Southern Atlantic	**0.108 (0.006)**	**0.001 (0.028)**
Western Atlantic vs. Eastern Atlantic	0.032 (0.168)	**0.001 (0.006)**

*Note:* Location Hierarchical Groupings: Northern Atlantic: western North Atlantic (WNA), Gulf of Mexico and Isla Mujeres, Mexico (GoMex), and eastern North Atlantic (ENA); Southern Atlantic: western South Atlantic (WSA), central Atlantic (ECA*), and eastern South Atlantic (ESA*); Western Atlantic: WNA, GoMex, and WSA; Eastern Atlantic: ENA, ECA*, and ESA*. * denote samples from these a priori subpopulations analyzed for SNPs only. Values in bold (mitogenome and SNP) indicate statistical significance at *p* < 0.05.

**FIGURE 2:**
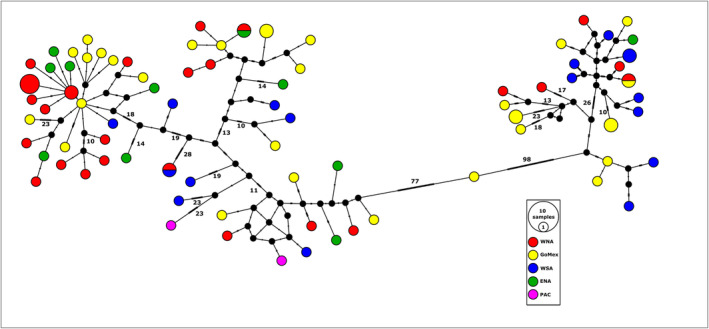
Median‐joining network of 90 shortfin mako shark complete mitogenome sequences collected from five areas within the Atlantic Ocean, with two Pacific mako shark mitogenomes as outgroups. Abbreviations and associated data: WNA, western North Atlantic; GoMex, Gulf of Mexico and Isla Mujeres, Mexico; WSA, western South Atlantic; ENA, eastern North Atlantic; PAC, Pacific Ocean. Numbers indicate mutational steps between haplotypes—only mutational steps ≥ 10 are numbered. Hatch marks represent single mutations and black nodes represent intermediate and unsampled sequences.

The median‐joining network for mitogenomes revealed a high number of mutational steps separating many of the haplotypes (up to 98 mutations), but little, if any, phylogeographic partitioning (Figure 2), indicating the presence of deep or long‐standing lineages that appear to be broadly sympatric within the Atlantic.

### 3.2 Single Nucleotide Polymorphisms

We obtained 1.145 billion PE reads across all 144 genotyped mako samples. However, many Reverse reads were of poor quality (per FastQC analysis); therefore, only Forward reads were used for downstream analyses. Stacks de novo assembly and filtering identified 168,339 genotyped SNPs across 144 samples. Secondary filtering using vcftools reduced the number of genotyped SNPs to 5202 sites across 115 individuals (see Appendix S1: Table S4 for results of step‐by‐step filtering as outlined in the Section 2). Following removal of duplicate samples (i.e., technical replicates only, *n* = 9) and one sample with missing capture coordinates, the mako SNP dataset comprised 5202 SNPs genotyped at 105 individuals. As kinship analysis identified only one putative‐related pair as 2nd‐degree relatives or higher (OC‐318 and OC‐363; kinship = 0.127) within the dataset, the multi‐locus genotypes of both sharks were retained.

**FIGURE 3:**
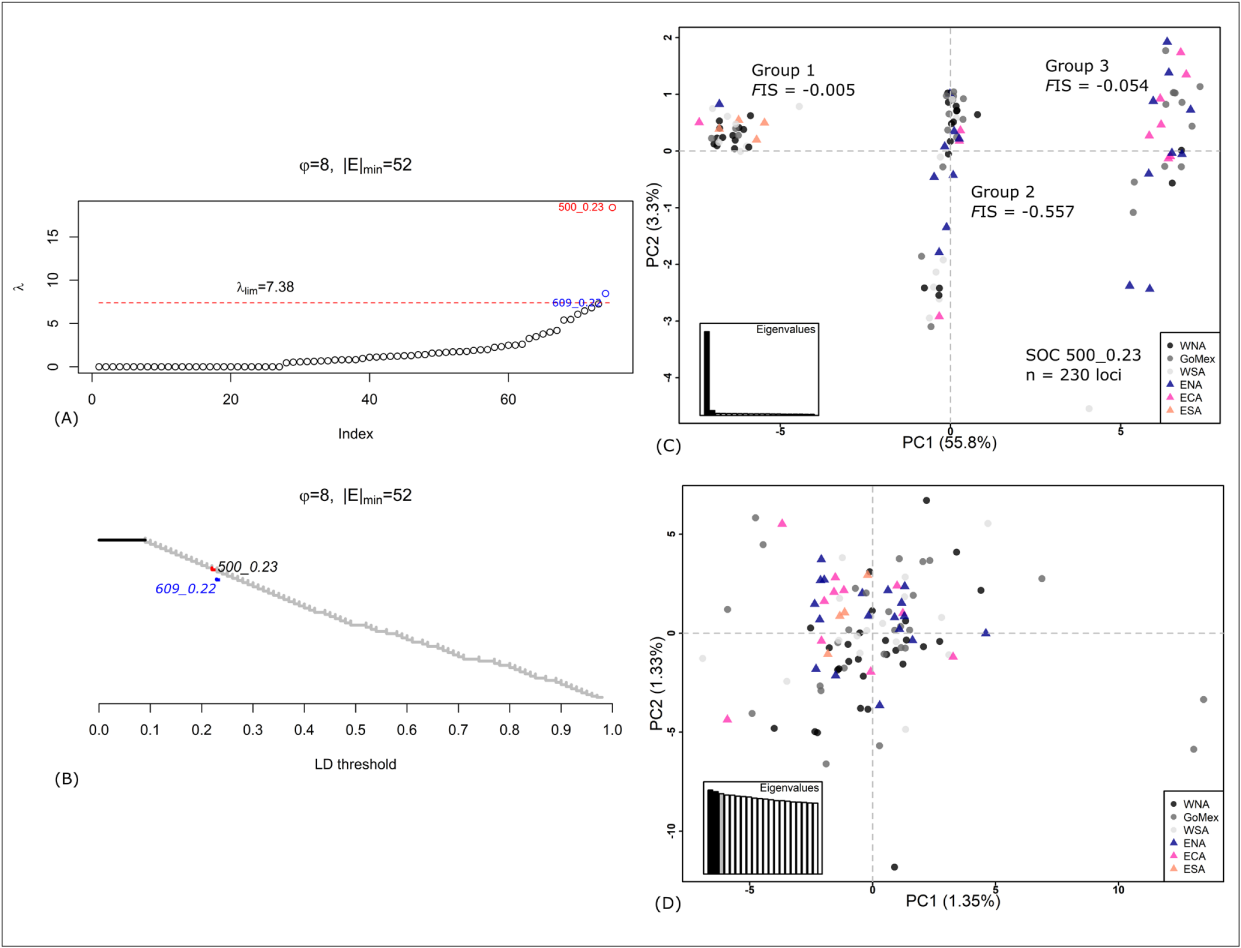
Visualization of the linkage disequilibrium network analysis (LDna) of shortfin mako shark RAD sequence data: (A) biplot depicting λ values (where λ is the change in the median linkage disequilibrium (LD) between loci in a cluster before and after its merger with a secondary cluster); outlier clusters (groups of tightly linked loci possessing very high λ values relative to the tree‐wide λ) are shown and appear above the lambda threshold (λ_lim_). Single outlier clusters (SOC) are shown in red; Compound outlier clusters (COC; i.e., a nested linkage group of loci) are shown in blue; (B) tree depicting pairwise *r* ^2^ values from 5202 genotyped SNPs, with branches corresponding to the single outlier cluster (SOC) and compound outlier cluster (COC) indicated in red and blue, respectively; (C) biplot displaying the first two principal components (PC; *x*‐axis = component 1, *y*‐axis = component 2) of a Principal Component Analysis (PCA) of 105 shortfin mako sharks genotyped at 230 single nucleotide polymorphisms (SNPs) identified by the LDna as a SOC (500_0.23). Inbreeding coefficients (*F*_IS_) for each of the three identified PCA clusters (Groups 1–3) are depicted; (D) biplot displaying the first two PCs of a PCA of 105 samples of shortfin mako sharks genotyped at 4971 neutral SNP loci. Location Abbreviations: WNA, western North Atlantic; GoMex, Gulf of Mexico and Isla Mujeres, Mexico; WSA, western South Atlantic; ENA, eastern North Atlantic; ECA, eastern Central Atlantic; ESA, eastern South Atlantic.

LDna identified two outlier clusters (1 SOC and 1 COC) within the mako RAD dataset (Figure 3): SOC 500_0.23 contained 230 tightly linked loci, while the compound outlier cluster (COC), 609_0.22, contained the previously defined SOC along with 52 additional SNP loci (i.e., 282 SNP loci total) (Appendix S1: Table S5, Figure S1; Figure 3A,B). Subsequent PCA of the 230 identified SOC SNP loci resolved three distinct clusters that separated along the first axis of the principal component—representing 55.8% of the total variance (Figure 3C); the second and third axes combined explained only 4.7% of the total genetic variance. Despite the presence of three distinct clusters in the PCA, no clear geographic partitioning of sharks was found [Figure 3C: Group 1 (*n* = 30): WNA = 13, GoMex = 3, WSA = 8, ENA = 1, ECA = 1, ESA = 4; Group 2 (*n* = 44): WNA = 13, GoMex = 12, WSA = 8, ENA = 8, ECA = 3, ESA = 0; Group 3 (*n* = 31): WNA = 2, GoMex = 12, WSA = 1, ENA = 9, ECA = 7, ESA = 0]. Furthermore, post hoc HWE testing (Wigginton, Cutler, and Abecasis 2005) of the frequency of each genotype (A/A, A/B, B/B) found no significant deviations from equilibrium expectations (*p* = 0.118).

Estimates of genetic diversity varied across the three SOC PCA groups: Groups 1 and 3 showed similar heterozygosity values and possessed *F*_IS_ estimates near zero (Group 1: *H*_O_ = 0.166; *H*_E_ = 0.162; *F*_IS_ = −0.005; Group 3: *H*_O_ = 0.157; *H*_E_ = 0.149; *F*_IS_ = −0.005), while members of Group 2 showed a large observed heterozygosity excess (*H*_O_ = 0.593; *H*_E_ = 0.381; *F*_IS_ = −0.557). Mapping of SOC consensus locus sequences (File S1: Table S1) to the only available (currently unannotated) reference genome for the mako (see Stanhope et al. 2023) yielded a high assignment of mako loci to a single contig, with 211 of the 230 consensus loci mapping to contig JANJGN010000001.1 with a percent identity ≥ 89.7% (File S1: Table S2). Assignments spanned 220,000,000 bp of scaffolded assembly (from 1,732,317 bp–221,412,477 bp), and notably, 206 SOC consensus sequences matched to this same contig with much higher precision (≥ 97% percent identity; File S1: Table S2). Matching of mako SOC consensus sequences using NCBI's Blastn feature to the white shark annotated reference genome matched 194 SOC consensus loci to a single white shark chromosome (NC_054467.1; Chromosome 1) with extremely low Expect values (1.00E‐47—8.00E‐74; File S1: Table S3).

Complementing LDna outcomes, BayeScan identified 68 candidate outlier SNPs, while OutFLANK identified 122. Across all three methods, the same 68 loci were deemed candidate outliers, while 106 loci were identified as candidates using both network analysis and OutFLANK; notably, there were no candidate outlier loci identified by both BayeScan and OutFLANK that were not also identified by LDna. As genomic features such as chromosomal inversions may present as erroneous population substructure (McKinney et al. 2020), we elected to remove all 230 SOC‐identified loci to ensure that downstream population genetic analyses were conducted only with putatively neutral loci that sort independently. After the SOC loci were removed from the dataset, 4972 putatively neutral SNP loci remained for downstream population genetic structure analyses. A single additional locus was discarded as it did not conform to HWE in two more subpopulations after FDR correction, leaving a final dataset containing 4971 mako SNP loci genotyped across 105 sharks (Table 1; Figure 1). Genetic diversity estimates were similar across Atlantic a priori subpopulations, and no private alleles within subpopulations were identified (Table 1). When sharks were grouped hierarchically (Western Atlantic versus Eastern Atlantic; Northern Atlantic versus Southern Atlantic), some evidence of genetic diversity variation was found: 19 private alleles were detected within mako sharks in the Western Atlantic (*n* = 72), with none detected in Eastern Atlantic sharks (*n* = 33) (although we note some sample size variation between these groups); 18 private alleles were detected within the Northern Atlantic sharks (*n* = 73), while none were found in Southern Atlantic sharks (*n* = 32).

Overall Atlantic‐wide genetic differentiation as measured by *F*_ST_ was low, but statistically significant (*F*_ST_ = 0.001; *p* = 0.023). No pairwise a priori subpopulation comparisons were statistically significant at *p* < 0.05 after FDR correction, but three were significant prior to FDR correction (Table 2). When samples were grouped hierarchically, the SNP data revealed very low, but statistically significant genetic differentiation between Western Atlantic versus Eastern Atlantic mako sharks (*F*_ST_ = 0.001, *p* = 0.006), but also between Northern Atlantic versus Southern Atlantic sharks (*F*_ST_ = 0.001, *p* = 0.028; Table 3).

**FIGURE 4:**
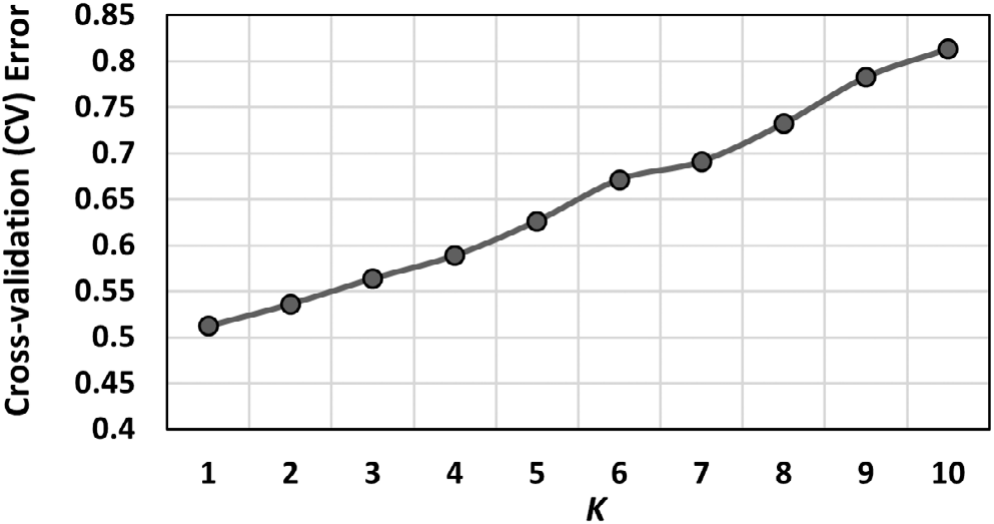
Plot of Admixture's (Alexander, Novembre, and Lange 2009) cross‐validation error values vs. number of clusters (*K*) for *K* = 1–10 for 105 shortfin mako samples genotyped at 4971 putative neutral SNP loci.

Individual‐based cluster analyses of the 4971 putatively neutral loci found no evidence of population genetic structure. The first three components of the PCA represented 3.97% of the total genetic variance in the SNP dataset and visualization of the first two components showed no marked clustering of individuals (Figure 3D). Likewise, Admixture's CV identified *K* = 1 (CV = 0.512) as the most appropriate number of genetic clusters within the mako SNP dataset (Figure 4).

Three of four surveyed hierfstat metrics showed some evidence of male‐biased dispersal: The overall *F*_ST_ for male makos (*n* = 47; *F*_ST_ = 0.000) was lower and significantly different than for female sharks (*n* = 58, *F*_ST_ = 0.002; *p* = 0.032). Likewise, the overall *F*_IS_ value was higher for males than females (*F*_IS_‐male = 0.019; *F*_IS_‐female = 0.016), and the mAIC value was lower for males than females (mAIC‐male = −3.616, mAIC‐female = 2.930); however, no statistical significance was found across the *F*_IS_ and mAIC tests (*p* > 0.05). In contrast, the vAIC value of female makos was higher than for males (but not statistically significant at *p* > 0.05).

## 4 Discussion

Global overfishing of pelagic sharks has resulted in the majority of species now threatened with extinction (Dulvy et al. 2008; Pacoureau et al. 2021). Securing species recovery requires urgent conservation management planning, which will be aided by a genomics‐scale view of the population biology of these species. Herein, we provide the first such population view of the Endangered (IUCN Red List) mako shark across the Atlantic Ocean. The high‐resolution, bi‐organelle, genomics assessment (5202 SNPs and 86 complete mitogenomes) revealed: (1) mako sharks are characterized by high levels of Atlantic‐wide, nuclear gene flow, but show evidence of restricted matrilineal connectivity between Northern and Southern Hemispheres; (2) similar levels of genome‐wide, neutral genetic diversity in makos across the Atlantic, (3) a signal of high linkage disequilibrium among a set of SNP loci which downstream population genetic analyses suggest may be an Atlantic‐wide, chromosomal inversion; and (4) comparatively high mitochondrial genome diversity relative to other at‐risk elasmobranch species.

### 4.1 Population Genomic Structure: Nuclear and Mitochondrial Connectivity

The mako shark's continuous temperate and tropical distribution in the Atlantic Ocean, combined with its often long‐distance migratory behavior and ability to use diverse habitats from nearshore waters to open ocean regions, permits expansive movements and spatial connectivity (Vaudo et al. 2017; Byrne et al. 2019). With regard to nuclear (SNP) data, the mako samples we analyzed from the western Atlantic (i.e., western North Atlantic, Gulf of Mexico, and western South Atlantic; *n* = 17–28) spanned a latitudinal range of ~40°N to 20°S (Figure 1), with the distance between even the closest cross‐equatorial sampling locations (i.e., southern Gulf of Mexico and southern Brazil) spanning more than 8000 km. Despite this immense distance, coupled with the presence of the Amazon River outlet and the warm waters blanketing the equator—all known biogeographic barriers to gene flow for many marine species including elasmobranchs (Hirschfeld et al. 2021; Tosetto et al. 2022)—there was no evidence of nuclear genetic differentiation between mako subpopulations across these barriers in the western Atlantic, nor much difference in nuclear diversity estimates (Table 1). Likewise, no latitudinal nuclear genetic differentiation was found among subpopulations of eastern Atlantic makos despite sampling across ~30°N to 20°S latitude (we note that sample sizes genotyped here were comparatively smaller; *n* = 4–18). Results from hierarchical analyses of the hemispheric groupings of Northern Atlantic versus Southern Atlantic makos also suggested little to no nuclear differentiation exists across these hemispheres given the extremely low (albeit statistically significant) *F*_ST_ value of 0.001 obtained (Table 3). These nuclear (bi‐parental) SNP‐based, high connectivity findings between Northern and Southern Hemispheres contrast with current ICCAT management boundaries for Atlantic makos (shown in Figure 1), and the mitogenome sequence‐based findings of statistically significant, matrilineal genetic differentiation across these hemispheres (i.e., between WNA and WSA sharks; between ENA and WSA sharks prior to FDR correction). The high nuclear connectivity results also contrast with available tagging data which has yet to record trans‐equatorial movements of mako sharks tagged in the north or south Atlantic [see Vaudo et al. 2016; Kohler and Turner 2019; Santos et al. 2021; Gibson et al. 2021; *n.b*. telemetry data from one mako tagged in the central Atlantic revealed it crossing the equator (Santos et al. 2021)], although we note these tagging data don't include many adult mako sharks, which likely undertake longer distance migrations.

Contrasting patterns of genetic connectivity between mitochondrial and nuclear markers in elasmobranchs are often attributed to sex‐biased dispersal (i.e., differences in the migratory propensity between males and females; Phillips et al. 2021) as each of these markers possesses distinct inheritance patterns. In shark species, males are generally the more dispersive sex, while females often show regional and/or natal philopatry (*sensu* Chapman et al. 2015)—a behavior defined as when a wide‐ranging female returns to the general region or specific location of her birth for parturition (Feldheim et al. 2014). As mitochondrial DNA is maternally transmitted in vertebrates, female‐only philopatric behavior over generations may drive the evolution of significant mitochondrial DNA differences among sharks born within geographically separate parturition habitats. Conversely, the bi‐parentally inherited nuclear DNA, which reflects the reproductive behaviors of both philopatric females and the more wide‐ranging, non‐philopatric males, may show negligible levels of genetic differentiation. Our findings of genomic population structure across the Northern and Southern Hemispheres that is evident in mitogenomes but not nuclear SNPs, combined with results of three of the four nuclear‐based, sex‐biased tests of dispersal (*F*_ST_, *F*_IS_ and mAIC), support the hypothesis of female philopatry and male‐mediated genetic dispersal, although female philopatry likely occurs at relatively large (hemispheric) spatial scales (see next paragraph). The genetic findings obtained here are also consistent with mako shark movements data available to date suggesting that adult males may be more dispersive than adult females (Gibson et al. 2021).

Fisheries catch records for mako sharks are consistent with the genetics‐based indications of female philopatry occurring at very broad, potentially hemispheric (western North vs. western South Atlantic) scales. First, pregnant makos have occasionally been caught in both north and south Atlantic waters, suggesting that parturition likely occurs in both hemispheres (Garcia‐Cortés et al. 2021); and second, catch data within the western North Atlantic show that the distribution of neonate and Young‐of‐Year (YOY) sharks is geographically widespread, spanning thousands of kilometers from the northern Gulf of Mexico to Newfoundland, Canada (Natanson et al. 2020), rather than concentrated within a few geographically distinct insular or neritic areas, lending weight to the hypotheses that female makos give birth across large, continuous swaths of habitat. Combined, these male and female movement and reproductive behaviors may be responsible for the: (1) nuclear homogeneity observed across this species' vast latitudinal range, (2) the mostly frequency‐based mitogenomic differentiation observed between Atlantic Northern and Southern Hemisphere mako sharks, and (3) the absence of distinct Atlantic phylogeographic matrilines (Figure 2).

Our mitogenomes‐based inferences verify the need for continued management by ICCAT of North and South Atlantic mako stocks separately, and verify the findings of the two other Atlantic mako mitochondrial DNA population studies conducted thus far, which were based on analyzing makos from fewer Atlantic regions and utilized short sections only of mitochondrial DNA (Heist, Musick, and Graves 1996; González et al. 2023). Interestingly, Corrigan et al. (2018), assessing mitochondrial complete control region sequences from makos across the Indo‐Pacific, also reported frequency‐based matrilineal differentiation between Northern and Southern Hemispheres in these ocean basins, with no phylogeographic partitioning. As in Atlantic Ocean waters, female parturition philopatry over even large Indo‐Pacific geographic scales would allow for some degree of frequency‐based matrilineal differentiation to evolve between hemispheres.

The population‐level pairwise analysis results obtained from SNP data showing very low but statistically significant genetic differentiation between some western and eastern Atlantic mako subpopulations in the Northern Hemisphere, albeit before FDR correction (Table 2), along with the presence of 19 private alleles within western sharks, were inconsistent with two other lines of evidence regarding population genetic differentiation. First, both individual‐based cluster analyses used showed no evidence of mako population structure in the Atlantic. Second, conventional tagging and satellite telemetry studies have documented recapture of mako sharks initially tagged off the Northeast USA in both the mid‐Atlantic and coastal waters off Portugal, Morocco, and the western Sahara (Kohler and Turner 2019; Byrne et al. 2019), thus indicating at least some spatial mixing is occurring across the Northern Atlantic. These conventional tagging data also indicate that the waters immediately west of the Azores may serve as a mixing area for western and eastern Atlantic makos (Kohler and Turner 2019).

Given the results from the individual‐based cluster analyses and long‐range mako tracking data, the reason for the population‐level analysis‐based, western vs. eastern North Atlantic population differentiation indicated by SNP markers remains enigmatic. However, if this statistical differentiation reflects a true biological signal, a speculative explanation, based on the occurrence of pregnant makos (Garcia‐Cortés et al. 2021) and evidence of parturition on both sides of the North Atlantic (Maia et al. 2007; Kohler and Turner 2019), is that there are multiple breeding populations, which could lead to subtle nuclear differentiation and the potential for longitudinal mako shark population structure across the Atlantic Ocean. However, drawing fisheries management recommendations based on the very low levels of genetic differentiation observed here is uncertain. To confirm our findings of nuclear genetic differentiation across the North Atlantic but absence of nuclear genetic structure between Northern and Southern Hemisphere collections, we recommend the targeted sampling and genomic analyses of mako shark neonates and YOY, since for many vagile species, signals of population genetic structure are most prominent when samples are obtained from these demographic groups which often show at least some short‐term residency or fidelity to their birth places (Klein et al. 2019).

### 4.2 Genomic Structural Variants and Areas of Reduced Nuclear Recombination

Recent work indicates chromosomal structural variants may play an important role in a number of biological and evolutionary processes, such as adaptation and speciation (Wellenreuther and Bernatchez 2018; Wellenreuther et al. 2019 and references therein), and that linkage disequilibrium network analysis (LDna) can identify genomic areas of low recombination and putative chromosomal inversions even across highly divergent taxa (e.g., Kemppainen et al. 2015—*Anopheles baimaii* and *Gasterosteus aculeatus* ; Jiménez‐Mena et al. 2020— *Ammodytes marinus* ; Hollenbeck et al. 2022— *Pecten maximus* ; Hearn et al. 2022— *Littorina saxatilis* ). Following network analysis of mako SNPs, multivariate PCA visualization of the 230 LDna‐identified SOC loci yielded three distinct sample clusters separated along the 1st principal component with no clear geographic partitioning (Figure 3C). This pattern of three distinct sample clusters—when associated with the intermediate group possessing a highly negative inbreeding coefficient (*F*_IS_) relative to the other two sample groupings—is suggestive of a chromosomal inversion (see Kemppainen et al. 2015 and examples therein). Thus, in Figure 3C, Groups 1 and 3 in the PCA likely contain sharks possessing either the ancestral or inverted karyotypes (e.g., A/A, and B/B), while Group 2 consists of sharks possessing the hetero‐karyotype (e.g., A/B), with each individual possessing one copy of the ancestral allele and one copy of the derived inverted chromosomal segment. Supporting this inference is the highly negative estimated *F*_IS_ value (−0.557) of the putative hetero‐karyotype grouping (Group 2), indicating the presence of an observed heterozygosity excess with respect to HWE proportions, while the *F*_IS_ values of sharks in Groups 1 and 3 were close to zero suggesting neither an excess nor a deficit. Similarly, the high map correspondence (> 89% identity) of most of the 230 SOC consensus sequences of ~140‐bp each (i.e., 211 of 230; ~92%) to a single, albeit very large contig (~220 million bp) from the mako genome reference scaffold assembly (Stanhope et al. 2023) supports the hypothesis that these loci may belong to a distinct region of low recombination in the genome. Likewise, using NCBI's Blastn feature, ~84% (194 of 230) of the SOC loci matched sequences on Chromosome 1 (NC_054467.1) of the white shark genome with a high percent identity (> 95%; and one of the three top Blastn matches) rather than to sequences scattered across the white shark's 42 nuclear chromosomes (File S1: Table S3). Where corresponding human NCBI Entrez Gene IDs could be found for white shark SOC gene homologs, these genomic features were largely involved in biological regulation (~76%): cellular processes (~69%), response to stimulus (~25%), and developmental and metabolic processes (~18% each). When we mapped our consensus loci to the white shark genome, akin to Domingues et al. (2022) one mako locus (62350_31) matched to the white shark locus “grid2,” a gene these authors designated as involved with development and nervous system function; herein, our search in the PANTHER database for its human homolog indicated involvement of this gene in biological regulation and cellular processes (File S1: Table S3).

As chromosomal inversions can suppress recombination, these structural variants may allow for the evolution, and ultimately inheritance, of a set of co‐adapted, tightly linked alleles, which may ultimately drive genetic differentiation between and/or within species. Makos are broadly distributed throughout the world's oceans, inhabit a diverse range of marine habitats (e.g., open ocean, neritic, and surface waters to depths of ~1000 m; Santos et al. 2021), and possess a wide thermal tolerance (5.2°C–31.1°C; Vaudo et al. 2016). However, to date, there are no data to suggest that some makos are locally or behaviorally adapted to specific geographic regions, and thus we are unable to link this putative chromosomal inversion to any sort of ecological adaptation. Indeed, we found no geographic partitioning of the candidate ancestral and inverted alleles, as each karyotype was found within the Western, Eastern, Northern, and Southern waters of the Atlantic and ocean‐wide; these genotypes (A/A, A/B, B/B) conformed HWE proportions, suggesting that if this pattern corresponds to a chromosomal inversion, this structural variant is panmictic throughout Atlantic Ocean makos. Nevertheless, since our tissue samples were obtained from mostly large juvenile and young adult sharks, rather than YOY and neonate sharks, it remains possible that the frequency of these inversion karyotypes varies among parturition areas and may be linked to yet unknown regional adaptive processes.

As our study was focused on the Atlantic Ocean, it is unknown if the three putative SOC karyotypes occur in makos globally, or if one or more of these genetic variants has become fixed within a discrete oceanic region. For instance, in their population genomic survey of the king scallop ( *Pecten maximus* ), Hollenbeck et al. (2022) identified three putative chromosomal inversions whose frequency was associated with variation in surface temperatures, with accompanying low levels of neutral differentiation throughout the northeast Atlantic. Similarly, Jiménez‐Mena et al. (2020) found high genetic connectivity in the lesser sandeel ( *Ammodytes marinus* ) within the North Sea using more than 2500 SNPs, but also found an SOC cluster of 13 SNPs which they identified as a putative structural variant, and whose karyotype spatial distribution indicated at least partial reproductive isolation of lesser sandeels within the Scottish North Sea coast. These examples suggest that if the frequency of the mako putative inversion varies globally, this structural variant may prove useful in helping to identify: (1) areas of restricted gene flow, (2) possible historical dispersal pathways and phylogeographic signals, and (3) areas of contemporary admixture or secondary‐contact zones of this species across its global range.

### 4.3 Genetic Diversity and Concluding Remarks

Conserving genetic diversity within species and their populations is of paramount importance to support their persistence in the Anthropocene, as genetic diversity underpins evolutionary fitness, adaptive potential, and resilience (DeWoody et al. 2021; Kardos et al. 2021; Hoban et al. 2023; Schmidt, Hoban, and Jetz 2023). While there is some debate concerning the most appropriate genetic targets for conservation focus (i.e., genome‐wide diversity vs. adaptative differentiation; e.g., see Kardos et al. 2021 and Teixeira and Huber 2021), a holistic approach is likely the most appropriate method to ensure the long‐term persistence of species across evolutionary timescales, as genome‐wide, neutral genetic diversity has been found to be correlated with functional and potentially adaptive evolutionary potential (Funk et al. 2012; DeWoody et al. 2021; Willi et al. 2022).

The similar estimates obtained in our study for genome‐wide, neutral genetic diversity in mako sharks across the Atlantic may either reflect high levels of gene flow, or that a disproportionate level of population reduction has not occurred in any specific region from heavy fisheries exploitation. Although genetic diversity estimates among panels of nuclear SNPs are not necessarily directly comparable across studies due to the diverse assembly and filtering strategies used and potential differences in chromosomal locations of the SNPs, the overall expected heterozygosity values we found (0.28) were relatively close to the estimates (0.341) reported for mako sharks by Domingues et al. (2022; albeit based on a smaller number of sharks (*n* = 22) and using 405 SNPs), indicating some concordance in the overall Atlantic mako shark genetic diversity results. A very tentative‐view comparison to genetic diversity estimates from SNP data on other pelagic, large‐bodied, highly migratory shark species that have similar global distributions and are also experiencing very high fisheries exploitation indicates genetic diversity in Atlantic mako sharks may be greater (mako shark: *H*_E_ = 0.176–0.250; *H*_O_ = 0.273–0.299; c.f. tiger shark: *H*_E_: 0.24–0.26; *H*_O_: 0.25–0.26, Bernard et al. 2021; blue shark: *H*_O_ = 0.139–0.145, *H*_E_ = 0.157–0.168; Nikolic et al. 2023).

In contrast to SNP data, sequence variability among mitogenomes is directly comparable across studies and species. To date, relatively few elasmobranch studies have assessed matrilineal population structure and diversity using a mitogenome sequence approach, but where data exist for comparison, mako shark mitogenome nucleotide diversity was equal or up to 34 times higher than other elasmobranch species, regardless sampled geographic range (Appendix S1: Table S6). The mitogenome and potentially nuclear genomic diversity findings found herein suggest that despite substantial overexploitation‐driven population declines, Atlantic makos appear to have retained a reasonable level of overall genetic diversity. This genetic status provides cautious optimism that this large‐bodied apex predator, which likely plays an influential oceanic ecosystem role, may still possess the genetic resilience and potential for adaptation to allow recovery in a rapidly changing ocean environment—if additional and/or enforced conservation management efforts are implemented to reduce overfishing. With increasing loss of species genetic diversity in the Anthropocene (Exposito‐Alonso et al. 2021), and assessments and monitoring of genetic diversity now called for by the United Nations Convention on Biological Diversity as important activities to inform national policy actions for conserving and restoring species (Hoban et al. 2023; Schmidt, Hoban, and Jetz 2023), the genetic diversity data we report here for mako sharks provide a baseline for assessing, and future monitoring, of the genetic health of this iconic species throughout its global distribution.

## Conflicts of Interest

The authors declare no conflicts of interest.

## Supporting information


Appendix S1.

File S1.

## Data Availability

All mitochondrial data underlying our analyses can be accessed at https://www.ncbi.nlm.nih.gov/ (GenBank Accession Numbers: PQ249310‐ PQ249394). Raw SNP data underlying our analyses have been deposited in Dryad: https://doi.org/10.5061/dryad.2280gb63f.

## References

[eva70071-bib-0001] Alexander, D. H. , J. Novembre , and K. Lange . 2009. “Fast Model‐Based Estimation of Ancestry in Unrelated Individuals.” Genome Research 19, no. 9: 1655–1664. 19648217 10.1101/gr.094052.109 PMC2752134

[eva70071-bib-0002] Andrews, S. 2010. “FastQC: A Quality Control Tool for High Throughput Sequence Data.” http://www.bioinformatics.babraham.ac.uk/projects/fastqc/.

[eva70071-bib-0003] Archer, F. I. , P. E. Adams , and B. B. Schneiders . 2017. “STRATAG: An R Package for Manipulating, Summarizing and Analyzing Population Genetic Data.” Molecular Ecology Resources 17, no. 1: 5–11. 10.1111/1755-0998.12559. 27327208

[eva70071-bib-0004] Bandelt, H. , P. Forster , and A. Röhl . 1999. “Median‐Joining Networks for Inferring Intraspecific Phylogenies.” Molecular Biology and Evolution 16, no. 1: 37–48. 10.1093/oxfordjournals.molbev.a026036. 10331250

[eva70071-bib-0005] Benjamini, Y. , and Y. Hochberg . 1995. “Controlling the False Discovery Rate: A Practical and Powerful Approach to Multiple Testing.” Journal of the Royal Statistical Society, Series B 57: 289–300. 10.1111/j.2517-6161.1995.tb02031.x.

[eva70071-bib-0006] Bernard, A. M. , K. A. Feldheim , M. R. Heithaus , S. P. Wintner , B. M. Wetherbee , and M. S. Shivji . 2016. “Global Population Genetic Dynamics of a Highly Migratory, Apex Predator Shark.” Molecular Ecology 21: 5312–5329. 10.1111/mec.13845. 27662523

[eva70071-bib-0007] Bernard, A. M. , K. A. Finnegan , P. P. Bitar , M. J. Stanhope , and M. S. Shivji . 2021. “Genomic Assessment of Global Population Structure in a Highly Migratory and Habitat Versatile Apex Predator, the Tiger Shark ( *Galeocerdo cuvier* ).” Journal of Heredity 112, no. 6: 497–507. 10.1093/jhered/esab046. 34374783

[eva70071-bib-0008] Bernard, A. M. , K. A. Finnegan , T. T. Sutton , R. I. Eytan , M. D. Weber , and M. S. Shivji . 2022. “Population Genomic Dynamics of Mesopelagic Lanternfishes *Diaphus dumerilii* , Lepidophanes Guentheri, and *Ceratoscopelus warmingii* (Family: Myctophidae) in the Gulf of Mexico.” Deep Sea Research, Part I 185: 103786. 10.1016/j.dsr.2022.103786.

[eva70071-bib-0009] Bernard, A. M. , V. P. Richards , M. J. Stanhope , and M. S. Shivji . 2018. “Transcriptome‐Derived Microsatellites Demonstrate Strong Genetic Differentiation in Pacific White Sharks.” Journal of Heredity 109, no. 7: 771–779. 10.1093/jhered/esy045. 30204894

[eva70071-bib-0010] Blower, D. C. , J. M. Pandolfi , B. D. Bruce , M. C. Gomez‐Cabrera , and J. R. Ovenden . 2012. “Population Genetics of Australian White Sharks Reveals Fine‐Scale Spatial Structure, Transoceanic Dispersal Events and Low Effective Population Sizes.” Marine Ecology Progress Series 455: 229–244. 10.3354/meps09659.

[eva70071-bib-0011] Bohling, J. , M. Small , J. Von Bargen , A. Louden , and P. DeHaan . 2019. “Comparing Inferences Derived From Microsatellite and RADseq Datasets: A Case Study Involving Threatened Bull Trout.” Conservation Genetics 20: 329–342. 10.3389/fgene.2020.00218.

[eva70071-bib-0012] Bushnell, B. , J. Rood , and E. Singer . 2017. “BBMerge – Accurate Paired Shotgun Read Merging via Overlap.” PLoS One 12, no. 10: e0185056. 10.1371/journal.pone.0185056. 29073143 PMC5657622

[eva70071-bib-0013] Byrne, M. E. , E. Cortés , J. J. Vaudo , et al. 2017. “Satellite Telemetry Reveals Higher Fishing Mortality Rates Than Previously Estimated, Suggesting Overfishing of an Apex Marine Predator.” Proceedings of the Royal Society B: Biological Sciences 284: 20170658. 10.1098/rspb.2017.0658. PMC5563797 28768885

[eva70071-bib-0014] Byrne, M. E. , J. J. Vaudo , H. G. C. McN , M. W. Johnston , B. M. Wetherbee , and M. S. Shivji . 2019. “Behavioral Response of a Mobile Marine Predator to Environmental Variables Differs Across Ecoregions.” Ecography 42, no. 9: 1569–1578. 10.1111/ecog.04463.

[eva70071-bib-0015] Camargo, S. M. , R. Coelho , D. Chapman , et al. 2016. “Structure and Genetic Variability of the Oceanic Whitetip Shark, *Carcharhinus longimanus* , Determined Using Mitochondrial DNA.” PLoS One 11, no. 5: e0155623. 10.1371/journal.pone.0155623. 27187497 PMC4871334

[eva70071-bib-0016] Cardeñosa, D. , J. Hyde , and S. Caballero . 2014. “Genetic Diversity and Population Structure of the Pelagic Thresher Shark ( *Alopias pelagicus* ) in the Pacific Ocean: Evidence for Two Evolutionarily Significant Units.” PLoS One 9, no. 10: e110193. 10.1371/journal.pone.0110193. 25337814 PMC4206417

[eva70071-bib-0017] Catchen, J. , P. A. Hohenlohe , S. Bassham , A. Amores , and W. A. Cresko . 2013. “Stacks: An Analysis Tool Set for Population Genomics.” Molecular Ecology 22, no. 11: 3124–3140. 10.1111/mec.12354. 23701397 PMC3936987

[eva70071-bib-0018] Chang, C. H. , K.‐T. Shao , Y.‐S. Lin , A.‐Y. Tsai , P.‐X. Su , and H.‐C. Ho . 2015. “The Complete Mitochondrial Genome of the Shortfin Mako, *Isurus oxyrinchus* (Chondrichthyes, Lamnidae).” Mitochondrial DNA 26, no. 3: 475–476. 10.3109/19401736.2013.834430. 24047173

[eva70071-bib-0019] Chapman, D. D. , K. A. Feldheim , Y. P. Papastamatiou , and R. E. Hueter . 2015. “There and Back Again: A Review of Residency and Return Migrations in Sharks, With Impolications for Population Structure and Management.” Annual Review of Marine Science 7: 547–570. 10.1146/annurev-marine-010814-015730. 25251267

[eva70071-bib-0020] Clarke, C. R. , S. A. Karl , R. L. Horn , et al. 2015. “Global Mitochondrial DNA Phylogeoraphy and Population Structure of the Silky Shark, *Carcharhinus falciformis* .” Marine Biology 162: 945–955. 10.1007/s00227-015-2636-6.

[eva70071-bib-0021] Corrigan, S. , A. D. Lowther , L. B. Beheregaray , et al. 2018. “Population Connectivity of the Highly Migratory Shortfin Mako ( *Isurus oxyrinchus* ) and Implications for Management in the Southern Hemisphere.” Frontiers in Ecology and Evolution 6: 187. 10.3389/fevo.2018.00187.

[eva70071-bib-0022] Cronin, M. R. , J. E. Amaral , A. M. Jackson , J. Jacquet , K. L. Seto , and D. A. Croll . 2023. “Policy and Transparency Gaps for Oceanic Shark and Rays in High Seas Tuna Fisheries.” Fish and Fisheries 24, no. 1: 56–70. 10.1111/faf.12710.

[eva70071-bib-0023] Danecek, P. , A. Auton , G. Abecasis , et al. 2011. “The Variant Call Format and VCFtools.” Bioinformatics 27, no. 15: 2156–2158. 10.1093/bioinformatics/btr330. 21653522 PMC3137218

[eva70071-bib-0024] DeWoody, J. A. , A. M. Harder , S. Mathur , and J. R. Willoughby . 2021. “The Long‐Standing Significance of Genetic Diversity in Conservation.” Molecular Ecology 30: 4147–4154. 10.1111/mec.16051. 34191374

[eva70071-bib-0025] Domingues, R. R. , C. C. Bruels , O. B. F. Gadig , D. D. Chapman , A. W. S. Hilsdorf , and M. S. Shivji . 2019. “Genetic Connectivity and Phylogeography of the Night Shark (*Charcharhinus signatus*) in the Western Atlantic Ocean: Implications for Conservation Management.” Aquatic Conservation: Marine and Freshwater Ecosystems 29, no. 1: 102–114. 10.1002/aqc.2961.

[eva70071-bib-0026] Domingues, R. R. , V. A. Mastrochirico‐Filho , N. J. Mendes , et al. 2022. “Gene‐Associated Markers as a Genomic and Transcriptomic Resource for a Highly Migratory and Apex Predator Shark ( *Isurus oxyrinchus* ).” Marine Biology 169, no. 9: 109. 10.1007/s00227-022-04094-z.

[eva70071-bib-0027] Dudgeon, C. L. , D. Broderick , and J. R. Ovenden . 2009. “IUCN Classification Zones Concord With, but Underestimate, the Population Genetic Structure of the Zebra Shark *Stegostoma fasciatum* in the Indo‐West Pacific.” Molecular Ecology 18, no. 2: 248–261. 10.1111/j.1365-294X.2008.04025.x. 19192179

[eva70071-bib-0028] Dulvy, N. K. , J. K. Baum , S. Clarke , et al. 2008. “You Can Swim but You Can't Hide: The Global Status and Conservation of Oceanic Pelagic Sharks and Rays.” Aquatic Conservation: Marine and Freshwater Ecosystems 18: 459–482. 10.1002/aqc.975.

[eva70071-bib-0029] Ebert, D. A. , M. Dando , and S. Fowler . 2021. Sharks of the World: A Complete Guide, 608. Princeton, UK: Princeton University Press.

[eva70071-bib-0030] Edgar, R. C. 2004. “MUSCLE: Multiple Sequence Alignment With High Accuracy and High Throughput.” Nucleic Acids Research 35, no. 5: 1792–1797. 10.1093/nar/gkh340. PMC390337 15034147

[eva70071-bib-0031] Excoffier, L. , and H. E. L. Lischer . 2010. “Arlequin Suite ver 3.5: A New Series of Programs to Perform Population Genetics Analyses Under Linux and Windows.” Molecular Ecology Resources 10: 564–567. 10.1111/j.1755-0998.2010.02847.x. 21565059

[eva70071-bib-0032] Exposito‐Alonso, M. , T. R. Booker , L. Czech , et al. 2021. “Genetic Diversity Loss in the Anthropocene.” Science 377: 1431–1435. 10.1126/science.abn5642. 36137047

[eva70071-bib-0033] Favre, L. , F. Balloux , J. Goudet , and N. Perrin . 1997. “Female‐Biased Dispersal in the Monogamous Mammal *Crocidura russula* : Evidence From Field and Microsatellite Patterns.” Proceedings of the Royal Society B: Biological Sciences 269, no. 1378: 127–132. 10.1098/rspb.1997.0019. PMC1688234 9061966

[eva70071-bib-0034] Feldheim, K. A. , S. H. Gruber , J. D. Dibattista , et al. 2014. “Two Decades of Genetic Profiling Yields First Evidence of Natal Philopatry and Long‐Term Fidelity to Parturition Sites in Sharks.” Molecular Ecology 23, no. 1: 110–117. 10.1111/mec.12583. 24192204

[eva70071-bib-0035] Feutry, P. , P. M. Kyne , R. D. Pillans , X. Chen , G. J. P. Naylor , and P. M. Grewe . 2014. “Mitogenomics of the Speartooth Shark Challenges Ten Years of Control Region Sequencing.” BMC Evolutionary Biology 14: 232. 10.1186/s12862-014-0232-x. 25406508 PMC4245800

[eva70071-bib-0036] Foll, M. , and O. Gaggiotti . 2008. “A Genome‐Scan Method to Identify Selected Loci Appropriate for Both Dominant and Codominant Markers: A Bayesian Perspective.” Genetics 180: 977–993. 10.1534/genetics.108.092221. 18780740 PMC2567396

[eva70071-bib-0037] Fu, Y.‐X. 1997. “Statistical Tests of Neutrality of Mutations Against Population Growth, Hitchhiking and Background Selection.” Genetics 147: 915–925. 10.1093/genetics/147.2.915. 9335623 PMC1208208

[eva70071-bib-0038] Funk, W. C. , J. K. McKay , P. A. Hohenlohe , and F. W. Allendorf . 2012. “Harnessing Genomics for Delineating Conservation Units.” Trends in Ecology & Evolution 27, no. 9: 489–496. 10.1016/j.tree.2012.05.012. 22727017 PMC4185076

[eva70071-bib-0039] Garcia‐Cortés, B. , A. Ramos‐Cartelle , J. Mejuto , A. Carroceda , and J. Fernández‐Costa . 2021. “Biological Observations of Shortfin Mako ( *Isurus oxyrinchus* ) on Spanish Surface Longline Fishery Targeting Swordfish.” Collective Volume of Scientific Papers ICCAT 78, no. 4: 64–96.

[eva70071-bib-0040] Gibson, K. J. , M. K. Streich , T. S. Topping , and G. W. Stunz . 2021. “New Insights Into the Seasonal Movement Patterns of Shortfin Mako Sharks in the Gulf of Mexico.” Frontiers in Marine Science 8: 623104. 10.3389/fmars.2021.623104.

[eva70071-bib-0041] González, M. T. , N. V. Leiva , P. M. Zárate , and J. A. Baeza . 2023. “Regional (South‐Eastern Pacific Ocean) Population Genetics and Global Phylogeography of Two Endangered Highly Migratory Pelagic Sharks, the Blue Shark Prionace Glauca and Shortfin Mako *Isurus oxyrinchus* .” Aquatic Conservation: Marine and Freshwater Ecosystems 33: 1098–1115. 10.1002/aqc.3987.

[eva70071-bib-0042] González, M. T. , F. A. Sepúlveda , P. M. Zárate , and J. A. Baeza . 2021. “Regional Population Genetics and Global Phylogeography of the Endangered Highly Migratory Shark *Lamna nasus* : Implications for Fishery Management.” Aquatic Conservation: Marine and Freshwater Ecosystems 31, no. 3: 620–634. 10.1002/aqc.3455.

[eva70071-bib-0043] Gorman, J. , N. Marra , M. S. Shivji , and M. J. Stanhope . 2019. “The Complete Mitochondrial Genome of an Atlantic Ocean Shortfin Mako Shark, *Isurus oxyrinchus* .” Mitochondrial DNA Part B Resources 4, no. 2: 3642–3643. 10.1080/23802359.2019.1677524. 33366122 PMC7707436

[eva70071-bib-0044] Goudet, J. 2004. “Heirfstat, a Package for R to Compute and Test Hierarchical F‐Statistics.” Molecular Ecology Resources 5, no. 1: 184–186. 10.1111/j.1471-8286.2004.00828.x.

[eva70071-bib-0045] Goudet, J. , N. Perrin , and P. Waser . 2002. “Tests for Sex‐Biased Dispersal Using Bi‐Parentally Inherited Genetic Markers.” Molecular Ecology 11, no. 6: 1103–1114. 10.1046/j.1365-294X.2002.01496.x. 12030985

[eva70071-bib-0046] Green, M. E. , S. A. Appleyard , W. T. White , S. R. Tracey , M. R. Heupel , and J. R. Ovenden . 2022. “Updated Connectivity Assessment for the Scalloped Hammerhead ( *Sphyrna lewini* ) in Pacific and Indian Oceans Using a Multi‐Marker Genetic Approach.” Fisheries Research 251: 106305. 10.1016/j.fishres.2022.106305.

[eva70071-bib-0047] Hearn, K. E. , E. L. Koch , S. Stankowski , et al. 2022. “Differing Associations Between Sex Determination and Sex‐Linked Inversions in Two Ecotypes of *Littorina saxatilis* .” Evolution Letters 6–5: 358–374. 10.1002/evl3.295. PMC9554762 36254259

[eva70071-bib-0048] Heist, E. J. , J. A. Musick , and J. E. Graves . 1996. “Genetic Population Structure of the Shortfin Mako ( *Isurus oxyrinchus* ) Inferred From Restriction Fragment Length Polymorphism Analysis of Mitochondrial DNA.” Canadian Journal of Fisheries and Aquatic Sciences 53, no. 3: 583–588. 10.1139/f95-245.

[eva70071-bib-0049] Hirschfeld, M. , C. Dudgeon , M. Sheaves , and A. Barnett . 2021. “Barriers in a Sea of Elasmobranchs: From Fishing for Populations to Testing Hypotheses in Population Genetics.” Global Ecology and Biogeography 30: 2147–2163. 10.1111/geb.13379.

[eva70071-bib-0050] Hoban, S. , J. M. da Silva , A. Mastretta‐Yanes , et al. 2023. “Monitoring Status and Trends in Genetic Diversity for the Convention on Biological Diversity: An Ongoing Assessment of Genetic Indicators in Nine Countries.” Conservation Letters 20: e12953. 10.1111/conl.12953.

[eva70071-bib-0051] Hollenbeck, C. M. , D. S. Portnoy , D. Garcia de la serrana , T. Magnesen , I. Matejusova , and I. A. Johnston . 2022. “Temperature‐Associated Selection Linked to Putative Chromosomal Inversions in King Scallop ( *Pecten maximus* ).” Proceedings of the Royal Society B: Biological Sciences 289: 20221573. 10.1098/rspb.2022.1573. PMC9532988 36196545

[eva70071-bib-0052] Holmes, B. J. , S. M. Williams , N. M. Otway , et al. 2017. “Population Structure and Connectivity of Tiger Sharks ( *Galeocerdo cuvier* ) Across the Indo‐Pacific Ocean Basin.” Royal Society Open Science 4: 170309. 10.1098/rsos.170309. 28791159 PMC5541554

[eva70071-bib-0053] ICCAT . 2017. “Report of the 2017 ICCAT Shortfin Mako Assessment Meeting, Madrid, Spain 12–16, June 2017.” https://www.iccat.int/documents/meetings/docs/2017_sma_ass_rep_eng.pdf.

[eva70071-bib-0054] Iwasaki, W. , T. Fukunaga , R. Isagozawa , et al. 2013. “MitoFish and MitoAnnotator: A Mitochondrial Genome Database of Fish With an Accurate and Automatic Annotation Pipeline.” Molecular Biology and Evolution 30, no. 11: 2531–2540. 10.1093/molbev/mst141. 23955518 PMC3808866

[eva70071-bib-0055] Jiménez‐Mena, B. , A. Le Moan , A. Christensen , et al. 2020. “Weak Genetic Structure Despite Strong Genomic Signal in Lesser Sandeel in the North Sea.” Evolutionary Applications 13: 376–387. 10.1111/eva.12875. 31993083 PMC6976957

[eva70071-bib-0056] Johri, S. , M. P. Doane , L. Allen , and E. A. Dinsdale . 2019. “Taking Advantage of the Genomics Revolution for Monitoring and Conservation of Chondrichthyan Populations.” Diversity 11: 49. 10.3390/d11040049.

[eva70071-bib-0057] Jombart, T. 2008. “Adegenet: A R Package for the Multivariate Analysis of Genetic Markers.” Bioinformatics 24: 1403–1405. 10.1093/bioinformatics/btn129. 18397895

[eva70071-bib-0058] Jombart, T. , and I. Ahmed . 2011. “Adegenet 1.3‐1: New Tools for the Analysis of Genome‐Wide SNP Data.” Bioinformatics 27, no. 21: 3070–3071. 10.1093/bioinformatics/btr521. 21926124 PMC3198581

[eva70071-bib-0059] Juan‐Jordá, M. J. , H. Murua , H. Arrizabalaga , G. Merino , N. Pacoureau , and N. K. Dulvy . 2022. “Seventy Years of Tunas, Billfishes, and Sharks as Sentinels of Global Ocean Health.” Science 378, no. 6620: eabj0211. 10.1126/science.abj0211. 36356144

[eva70071-bib-0060] Junge, C. , S. C. Donnellan , C. Huveneers , et al. 2019. “Comparative Population Genomics Confirms Little Population Structure in Two Commercially Targeted Carcharhinid Sharks.” Marine Biology 166: 16. 10.1007/s00227-018-3454-4.

[eva70071-bib-0061] Kamvar, Z. N. , J. C. Brooks , and N. J. Grünwald . 2015. “Novel R Tools for Analysis of Genome‐Wide Population Genetic Data With Emphasis on Clonality.” Frontiers in Genetics 6: 208. 10.3389/fgene.2015.00208. 26113860 PMC4462096

[eva70071-bib-0062] Kamvar, Z. N. , J. F. Tabima , and N. J. Grünwald . 2014. “Poppr: An R Package for Genetic Analysis of Populations With Clonal, Partially Clonal, and/or Sexual Reproduction.” PeerJ 2: e281. 10.7717/peerj.281. 24688859 PMC3961149

[eva70071-bib-0063] Kardos, M. , E. E. Armstrong , S. W. Fitzpatrick , et al. 2021. “The Crucial Role of Genome‐Wide Genetic Variation in Conservation.” Proceedings of the National Academy of Sciences 118, no. 48: e2104642118. 10.1073/pnas.2104642118. PMC8640931 34772759

[eva70071-bib-0064] Kearse, M. , R. Moir , A. Wilson , et al. 2012. “Geneious Basic: An Integrated and Extendable Desktop Software Platform for the Organization and Analysis of Sequence Data.” Bioinformatics 28, no. 12: 1647–1649. 10.1093/bioinformatics/bts199. 22543367 PMC3371832

[eva70071-bib-0065] Kemppainen, P. 2014. LDna: Linkage Disequilibrum Network Analysis (LDna): An Introduction LDna. St. Murrieta, CA: Basics.

[eva70071-bib-0066] Kemppainen, P. , C. G. Knight , D. K. Sarma , et al. 2015. “Linkage Disequilibrium Network Analysis (LDna) Gives a Global View of Chromosomal Inversions, Local Adaptation and Geographic Structure.” Molecular Ecology Resources 15: 1031–1045. 10.1111/1755-0998.12369. 25573196 PMC4681347

[eva70071-bib-0067] Klein, J. D. , A. E. Bester‐van der Merwe , M. L. Dicken , K. L. Mmonwa , and P. R. Teske . 2019. “Reproductive Philopatry in a Coastal Shark Drives Age‐Related Population Structure.” Marine Biology 166: 26. 10.1007/s00227-019-3467-7.

[eva70071-bib-0068] Knaus, B. J. , and N. J. Grünwald . 2017. “VCFR: A Package to Manipulate and Visualize Variant Call Format Data in R.” Molecular Ecology Resources 17, no. 1: 44–53. 10.1111/1755-0998.12549. 27401132

[eva70071-bib-0069] Kohler, N. E. , and P. A. Turner . 2019. “Distributions and Movements of Atlantic Shark Species: A 52‐Year Retrospective Atlas of Mark and Recapture Data.” Marine Fisheries Review 81, no. 2: 1–93. 10.7755/MFR.81.2.1.

[eva70071-bib-0070] Kraft, D. W. , E. E. Conklin , E. W. Barba , et al. 2020. “Genomics Versus mtDNA for Resolving Stock Structure in the Silky Shark ( *Carcharhinus falciformis* ).” PeerJ 8: e10186. 10.7717/peerj.10186. 33150082 PMC7585369

[eva70071-bib-0071] Leigh, J. W. , and D. Bryant . 2015. “PopART: Full‐Feature Software for Haplotype Network Construction.” Methods in Ecology and Evolution 6, no. 9: 1110–1116. 10.1111/2041-210X.12410.

[eva70071-bib-0072] Lotterhos, K. E. , and M. C. Whitlock . 2015. “The Relative Power of Genome Scans to Detect Local Adaptation Depends on Sampling Design and Statistical Method.” Molecular Ecology 24, no. 5: 1031–1046. 10.1111/mec.13100. 25648189

[eva70071-bib-0073] Lou, R. N. , N. K. Fletcher , A. P. Wilder , D. O. Conover , N. O. Therkildsen , and J. B. Searle . 2018. “Full Mitochondrial Genome Sequences Reveal New Insights About Post‐Glacial Expansion and Regional Phylogeographic Structure in the Atlantic Silverside ( *Menidia menidia* ).” Marine Biology 165, no. 8: 124. 10.1007/s00227-018-3380-5.

[eva70071-bib-0074] Maia, A. , N. Queiroz , H. N. Cabral , A. M. Santos , and J. P. Correia . 2007. “Reproductive Biology and Population Dynamics of the Shortfin Mako, *Isurus oxyrinchus* Rafinesque, 1810, off the Southwest Portuguese Coast, Eastern North Atlantic.” Journal of Applied Ichthyology 23: 246–251. 10.1111/j.1439-0426.2007.00849.x.

[eva70071-bib-0076] McKinney, G. , M. V. McPhee , C. Pascal , J. E. Seeb , and L. W. Seeb . 2020. “Network Analysis of Linkage Disequilibrium Reveals Genome Architecture in Chum Salmon.” G3: Genes, Genomes, Genetics 10, no. 5: 1553–1561. 10.1534/g3.119.400972. 32165371 PMC7202013

[eva70071-bib-0077] Mehlrose, M. R. , A. M. Bernard , K. A. Finnegan , L. E. Krausfeldt , J. V. Lopez , and M. S. Shivji . 2022. “Three Complete Mitochondrial Genomes of Shortfin Mako Sharks, *Isurus oxyrinchus* , From the Atlantic and Pacific Oceans.” Mitochondrial DNA Part B Resources 7, no. 4: 652–654. 10.1080/23802359.2022.2060768. 35434361 PMC9009892

[eva70071-bib-0078] Natanson, L. J. , M. Winton , H. Bowlby , et al. 2020. “Updated Reproductive Parameters for the Shortfin Mako ( *Isurus oxyrinchus* ) in the North Atlantic Ocean With Inferences of Distribution by Sex and Reproductive Stage.” Fishery Bulletin 118: 21–36.

[eva70071-bib-0079] Neph, S. , M. S. Kuehn , A. P. Reynolds , et al. 2012. “BEDOPS: High‐Performance Genomic Feature Operations.” Bioinformatics 28, no. 14: 1919–1920. 10.1093/bioinformatics/bts277. 22576172 PMC3389768

[eva70071-bib-0080] Nikolic, N. , F. Devloo‐Delva , D. Bailleul , et al. 2023. “Stepping up to Genome Scan Allows Stock Differentiation in the Worldwide Distributed Blue Shark *Prionace glauca* .” Molecular Ecology 32, no. 5: 1000–1019. 10.1111/mec.16822. 36511846

[eva70071-bib-0081] Pacoureau, N. , C. L. Rigby , P. M. Kyne , et al. 2021. “Half a Century of Global Decline in Oceanic Sharks and Rays.” Nature 589: 567–571. 10.1038/s41586-020-03173-9. 33505035

[eva70071-bib-0082] Paetkau, D. , W. Calvert , I. Stirling , and C. Strobeck . 1995. “Microsatellite Analysis of Population Structure in Canadian Polar Bears.” Molecular Ecology 4: 347–354. 10.1111/j.1365-294X.1995.tb00227.x. 7663752

[eva70071-bib-0083] Paradis, E. 2010. “Pegas: An R Package for Population Genetics With an Integrated–Modular Approach.” Bioinformatics 26: 419–420. 10.1093/bioinformatics/btp696. 20080509

[eva70071-bib-0084] Peterson, B. K. , J. N. Weber , E. H. Kay , H. S. Fisher , and H. E. Hoekstra . 2012. “Double Digest RADSeq: An Inexpensive Method for De Novo SNP Discovery and Genotyping in Model and Non‐model Species.” PLoS One 7, no. 5: e37135. 10.1371/journal.pone.0037135. 22675423 PMC3365034

[eva70071-bib-0085] Phillips, N. M. , F. Devloo‐Delva , C. McCall , and T. S. Daly‐Engel . 2021. “Reviewing the Genetic Evidence for Sex‐Biased Dispersal in Elasmobranchs.” Reviews in Fish Biology and Fisheries 31: 821–841. 10.1007/s11160-021-09673-9.

[eva70071-bib-0086] R Core Team . 2021. R: A Language and Environment for Statistical Computing. Vienna, Austria: R Foundation for Statistical Computing.

[eva70071-bib-0087] Rigby, C. L. , R. Barreto , J. Carlson , et al. 2019. “ *Isurus oxyrinchus* .” The IUCN Red List of Threatened Species 2019: e. T39341A2903170.

[eva70071-bib-0088] Rochette, N. C. , A. G. Rivera‐Colón , and J. M. Catchen . 2019. “Stacks 2: Analytical Methods for Paired‐End Sequencing Improve RADseq‐Based Population Genomics.” Molecular Ecology 28, no. 21: 4737–4754. 10.1111/mec.15253. 31550391

[eva70071-bib-0089] Ruck, C. L. , M. S. Shivji , R. W. Jabado , and A. M. Bernard . 2024. “Cross Ocean‐Basin Population Genetic Dynamics in a Pelagic Top Predator of High Conservation Concern, the Oceanic Whitetip Shark, *Carcharhinus longimanus* .” Conservation Genetics 25: 677–695. 10.1007/s10592-023-01596-1.

[eva70071-bib-0090] Santos, C. C. , A. Domingo , J. Carlson , et al. 2021. “Movements, Habitat Use, and Diving Behavior of Shortfin Mako in the Atlantic Ocean.” Frontiers in Marine Science 8: 6343. 10.3389/fmars.2021.686343.

[eva70071-bib-0091] Schmidt, C. , S. Hoban , and W. Jetz . 2023. “Conservation Macrogenetics: Harnessing Genetic Data to Meet Conservation Commitments.” Trends in Genetics 39: 816–829. 10.1016/j.tig.2023.08.002. 37648576

[eva70071-bib-0092] Schrey, A. W. , and E. J. Heist . 2003. “Microsatellite Analysis of Population Structure in Shortfin Mako.” Canadian Journal of Fisheries and Aquatic Sciences 60: 670–675. 10.1139/f03-064.

[eva70071-bib-0093] Sims, D. W. , G. Mucientes , and N. Queiroz . 2018. “Shortfin Mako Sharks Threatened by Inaction.” Science 359, no. 6382: 1342. 10.1126/science.aat0315. 29567698

[eva70071-bib-0094] Sims, D. W. , G. Mucientes , and N. Queiroz . 2021. “Shortfin Mako Sharks Speeding to the Brink.” Science 371, no. 6527: 355. 10.1126/science.abg2355. 33479143

[eva70071-bib-0095] Sort, M. , A. Manuzzi , B. Jiménez‐Mena , et al. 2021. “Come Together: Calibration of Tiger Shark ( *Galeocerdo cuvier* ) Microsatellite Databases for Investigating Global Population Structure and Assignment of Historical Specimens.” Conservation Genetics Resources 13: 209–220. 10.1007/s12686-021-01197-5.

[eva70071-bib-0096] Stanhope, M. J. , K. M. Ceres , S. Qi , et al. 2023. “Genomes of Endangered Great Hammerhead and Shortfin Mako Sharks Reveal Historic Population Declines and High Levels of Inbreeding in Great Hammerhead.” iScience 26, no. 1: 105815. 10.1016/j.isci.2022.105815. 36632067 PMC9826928

[eva70071-bib-0097] Taguchi, M. , T. Kitamura , and K. Yokawa . 2011. Genetic Population Structure of Shortfin Mako ( *Isurus oxyrinchus* ) Inferred From Mitochondrial DNA on Inter‐Oceanic Scale. Taipei, China: International Scientific Committee for Tuna and Tuna‐Like Species in the North Pacific Ocean, ISC/11/SHARKWG‐1/02.

[eva70071-bib-0098] Tajima, F. 1989. “Statistical Method for Testing the Neutral Mutation Hypothesis by DNA Polymorphism.” Genetics 123: 585–595. 10.1093/genetics/123.3.585. 2513255 PMC1203831

[eva70071-bib-0099] Teacher, A. G. , C. André , J. Merilä , and C. W. Wheat . 2012. “Whole Mitochondrial Genome Scan for Population Structure and Selection in the Atlantic Herring.” BMC Evolutionary Biology 12: 248. 10.1186/1471-2148-12-248. 23259908 PMC3545857

[eva70071-bib-0100] Teixeira, J. C. , and C. D. Huber . 2021. “The Inflated Significance of Neutral Genetic Diversity in Conservation Genetics.” Proceedings of the National Academy of Sciences USA 118: e2015096118. 10.1073/pnas.2015096118. PMC7958437 33608481

[eva70071-bib-0101] Thomas, P. D. , D. Ebert , A. Muruganujan , T. Mushayahama , L.‐P. Albou , and H. Mi . 2022. “PANTHER: Making Genome‐Scale Phylogenetics Accessible to all.” Protein Science 31, no. 1: 8–22. 10.1002/pro.4218. 34717010 PMC8740835

[eva70071-bib-0102] Thrasher, D. J. , B. G. Butcher , L. Campagna , M. S. Webster , and I. J. Lovette . 2018. “Double‐Digest RAD Sequencing Outperforms Microsatellite Loci at Assigning Paternity and Estimating Relatedness: A Proof of Concept in a Highly Promiscuous Bird.” Molecular Ecology Resources 18: 953–965. 10.1111/1755-0998.12771. 29455472

[eva70071-bib-0103] Tosetto, E. G. , A. Bertrand , S. Neumann‐Leitão , and M. N. Júnior . 2022. “The Amazon River Plume, a Barrier to Animal Dispersal in the Western Tropical Atlantic.” Scientific Reports 12: 537. 10.1038/s41598-021-04165-z. 35017566 PMC8752809

[eva70071-bib-0104] Vaudo, J. J. , M. E. Byrne , B. M. Wetherbee , G. M. Harvey , and M. S. Shivji . 2017. “Long‐Term Satellite Tracking Reveals Region‐Specific Movements of a Large Pelagic Predator, the Shortfin Mako Shark, in the Western North Atlantic Ocean.” Journal of Applied Ecology 54: 1765–1775. 10.1111/1365-2664.12852.

[eva70071-bib-0105] Vaudo, J. J. , B. M. Wetherbee , A. D. Wood , et al. 2016. “Vertical Movements of Shortfin Mako Sharks *Isurus oxyrinchus* in the Western North Atlantic Ocean Are Strongly Influenced by Temperature.” Marine Ecology Progress Series 547: 163–175. 10.3354/meps11646.

[eva70071-bib-0106] Vaux, F. , S. Bohn , J. R. Hyde , and K. G. O'Malley . 2021. “Adaptive Markers Distinguish North and South Pacific Albacore Amid Low Population Differentiation.” Evolutionary Applications 14, no. 5: 1343–1364. 10.1111/eva.13202. 34025772 PMC8127716

[eva70071-bib-0107] Vella, N. , and A. Vella . 2023. “Phylogeographic Analyses of the Shortfin Mako, *Isurus oxyrinchus* Rafinesque, 1810 (Chondrichthyes: Lamniformes) From the Central Mediterranean Sea, a Critically Endangered Species in the Region.” Fishes 8, no. 10: 520. 10.3390/fishes8100520.

[eva70071-bib-0108] Vendrami, D. L. J. , M. De Noia , L. Telesca , et al. 2019. “RAD Sequencing Sheds New Light on the Genetic Structure and Local Adaptation of European Scallops and Resolves Their Demographic Histories.” Scientific Reports 9: 7455. 10.1038/s41598-019-43939-4. 31092869 PMC6520335

[eva70071-bib-0109] Villesen, P. 2007. “FaBox: An Online Toolbox for Fasta Sequences.” Molecular Ecology Notes 7, no. 6: 965–968. 10.1111/j.1471-8286.2007.01821.x.

[eva70071-bib-0110] Weir, B. S. , and C. C. Cockerham . 1984. “Estimating F‐Statistics for the Analysis of Population Structure.” Evolution 38, no. 6: 1358–1370. 10.2307/2408641. 28563791

[eva70071-bib-0111] Wellenreuther, M. , and L. Bernatchez . 2018. “Eco‐Evolutionary Genomics of Chromosomal Inversions.” Trends in Ecology & Evolution 33, no. 6: 427–440. 10.1016/j.tree.2018.04.002. 29731154

[eva70071-bib-0112] Wellenreuther, M. , C. Mérot , E. Berden , and L. Bernatchez . 2019. “Going Beyond SNPs: The Role of Structural Variants in Adaptive Evolution and Species Diversification.” Molecular Ecology 28: 1203–1209. 10.1111/mec.15066. 30834648

[eva70071-bib-0113] Wigginton, J. E. , D. J. Cutler , and G. R. Abecasis . 2005. “A Note on Exact Tests of Hardy–Weinberg Equilibrium.” AJHG 76, no. 5: 887–893. 10.1086/429864. 15789306 PMC1199378

[eva70071-bib-0114] Willi, Y. , T. N. Kristensen , C. M. Sgrò , A. R. Weeks , M. Ørsted , and A. A. Hoffman . 2022. “Conservation Genetics as a Management Tool: The Five Best‐Supported Paradigms to Assist the Management of Threatened Species.” Proceedings of the National Academy of Sciences 119, no. 1: e2105076119. 10.1073/pnas.2105076119. PMC8740573 34930821

[eva70071-bib-0115] Wilson, C. C. , M. Lavender , and J. Black . 2007. “Genetic Assessment of Walleye ( *Sander vitreus* ) Restoration Efforts and Options in Nipigon Bay and Black Bay, Lake Superior.” Journal of Great Lakes Research 33, no. Supplement 1: 133–144.

[eva70071-bib-0116] Zheng, X. , D. Levine , J. Shen , S. M. Gogarten , C. Laurie , and B. S. Weir . 2012. “A High‐Performance Computing Toolset for Relatedness and Principal Component Analysis of SNP Data.” Bioinformatics 28, no. 24: 3326–3328. 10.1093/bioinformatics/bts606. 23060615 PMC3519454

[eva70071-bib-0117] Zimmerman, S. J. , C. L. Aldridge , and S. J. Oyler‐McCance . 2020. “An Empirical Comparison of Population Genetic Analyses Using Microsatellite and SNP Data for a Species of Conservation Concern.” BMC Genomics 21: 382. 10.1186/s12864-020-06783-9. 32487020 PMC7268520

