# Supplementary material for: Connections Across Open Water: A Bi‐Organelle, Genomics‐Scale Assessment of Atlantic‐Wide Population Dynamics in a Pelagic, Endangered Apex Predator Shark (Isurus oxyrinchus)
Source: Evol Appl. 2025 Jan 22;18(1):e70071. doi: 10.1111/eva.70071 (PMC11754249; doi:10.1111/eva.70071)
Supplement: Supplementary file 1 — Appendix S1. [file EVA-18-e70071-s002.docx]

**Supplemental Methods and Results**

**Supplemental Methods 1: Mitochondrial Long PCR**

Initial amplifications of the mitochondrial genome occurred in four fragments spanning 3796-5895 bp; however, when some of these fragment amplifications failed, they were divided into smaller sub-fragments (A and B; lengths 1323-3497 bp) for re-amplification. All PCR amplifications were performed using a combination of previously published primers and new mitochondrial DNA primers designed as necessary with the software Geneious Prime 2021.2.2 (https://www.geneious.com) with two complete shortfin mako mitogenomes as reference (GenBank Accession Numbers: KF361861.1, Chang et al. 2015; MF537044.1, Gorman et al. 2017) (Table S2).
 Fragments 1 through 4, and sub-fragments 1A-3B were PCR amplified in 25-μL reaction volumes containing 1-μL of template genomic DNA, 0.75-μL [10 μM] of each Forward and Reverse primer, 12.5-μL of KAPA HiFi HotStart ReadyMix (Roche Molecular Systems, Inc.; containing KAPA HiFi HotStart DNA Polymerase [0.5 U], buffer, dNTPs [0.3 mM of each dNTP], MgCl_2_ [2.5 mM]), and 10-μL of ddH_2_0. Fragments 1 through 4 were amplified in an Applied Biosystems BioRad thermocycler (Thermo Fisher Scientific, Inc.) via a touchdown protocol, which consisted of an initial activation period of 5 minutes (min) at 95°C, followed by five cycles of denaturation at 95°C for 60 seconds (sec), annealing at 65°C for 60 sec (decreasing by one degree Celsius with each successive cycle), and an extension at 72°C for 5 min, then an additional 30 cycles of denaturation at 95°C for 60 sec, annealing at 50°C for 60 sec, and elongation at 72°C for 30 sec were performed, with a final extension step of 10 min at 72°C. Sub-fragments 1A-3B were amplified with a thermal profile consisting of an initial activation at 95°C for 5 min, followed by 35 cycles of denaturation, annealing, and extension at 94°C (60 sec), 50°C (90 sec), and 72° (90 sec), respectively, with a final extension at 72°C for 20 min. Sub-fragments 4A and 4B were amplified in 20-μL reaction volumes containing 1-μL of template genomic DNA, 0.5-μL [10 μM] of each Forward and Reverse primer, 5-μL of QIAGEN LongRange PCR Kit Mix and 13-μL of ddH_2_0. PCR amplifications consisted of an initial activation at 93°C for 180 sec, followed by 35 cycles of denaturation, annealing, and extension at 93°C (30 sec), 52°C (15 sec), and 68° (120 sec), respectively, with a final extension at 72°C for 10 min. Amplification success of all fragments was determined by means of agarose gel (1.2%) electrophoresis. Where non-target amplification occurred (Sub-fragments 1A-3B), a QIAquick Gel Extraction Kit (QIAGEN, Inc.) was used according to manufacturer’s guidelines to isolate and purify the desired (appropriately sized) amplicon for downstream library preparation and sequencing. All other resultant amplicons (Fragments: 1-4, Sub-fragments: 4A and 4B) were purified using AMPure XP beads (Beckman Coulter Inc.) and eluted to a final volume of 75-µL using the QIAGEN Buffer EB. A Qubit 3 Fluorometer (Invitrogen) was used to quantify the final concentrations of all PCR products.

**Supplemental Methods 2: Mitochondrial DNA Sanger Sequencing Reaction Conditions and sequencing**
Sub-fragments 3B1-3B4, 4A1-4A5, and 4B1-4B4 were amplified in 25-μL reaction volumes, containing 0.75-μL [10 μM] of each Forward and Reverse primer (Table S2), 12.5-μL of KAPA HiFi HotStart ReadyMix (containing KAPA HiFi HotStart DNA Polymerase [0.5 U], buffer, dNTPs [0.3 mM of each dNTP], MgCl_2_ [2.5 mM]), and 10-μL of ddH_2_0. Thermal profiles varied among sub-fragments with respect to annealing temperature and duration; however, each consisted of an initial activation at 94°C for 180 sec, followed by 35 cycles of denaturation (94°C for 30 sec), annealing (50-57°C for 60-90 sec depending on primer combination), and extension (68°C for 120 sec). Each amplification possessed a final extension at 72°C for 5-10 min.

Size and quality of PCR amplifications were confirmed via electrophoresis on a 1.2% agarose gel. Enzymatic PCR purification and cycle sequencing (BigDye Terminator v3.1 Cycle Sequencing Kit) was performed by GENEWIZ, Inc. (South Plainsfield, NJ), with Sanger sequencing of resultant products on an AB3730xl DNA Analyzer. Mitochondrial DNA sequences were manually inspected to ensure proper base-calling, and sub-fragments were assembled into contigs in Geneious Prime using the ‘Map to Reference’, ‘Geneious’ Mapper, and reference sequence GB#: KF361861 (Chang et al. 2015). Following contig assembly, a consensus sequence was generated and aligned to the Illumina-derived consensus sequence to complete the mitogenome assembly.

**Supplemental Methods 3: Stacks *de novo* SNP Assembly and Parameter Optimization**

Demultiplexing, initial quality filtering, and recovery of barcodes and rad tags was performed using the stacks module *process_radtags* [options: -c, -q, -r, -E (option: phred33)], and reads were filtered for adapter sequences (option: adapter_1; 5’-AGATCGGAAGAG-3’) and truncated to 140 nucleotides in length. Optimization of Stacks parameters for *de novo* SNP assembly was completed as outlined in Rochette and Catchen (2017) and Paris et al. (2017) using a data subset (*n* = 12) and by exploring a range of values (1-9) for the parameters *M* (maximum number of nucleotide distance allowed between stacks within a single individual) and *n* (the number of nucleotide mismatches allowed between sample loci when building the locus catalog), where *M* = *n* across runs, and the parameter *m* (minimum stack depth coverage) was fixed at 3. Optimal Stacks parameter values (*m* = 3, *M* = 3, *n* = 3) for downstream *de novo* assembly were selected upon stabilization of the number of loci shared by 80% of samples and the number of SNPs per locus.

Following optimization of Stacks *de novo* assembly parameters, the module *process_radtags* was run as outlined above, followed by the module *ustacks*, assuming *m* = *M* = 3 and all other default settings. The Stacks catalog (*cstacks* module) was built using a subset of 14 shortfin mako samples and assuming the parameter *n* = 3. The modules *sstacks*, *tsv2bam* and *gstacks* were run using default settings and the raw variant dataset was filtered using the *populations* module to retain only those loci that: (1) occurred in all six subpopulations (-p = 6), (2) occurred in at least 20% of individuals per subpopulation (-r = 0.2), and (3) possessed a maximum observed heterozygosity of 0.6 (--max-obs-het = 0.6). The parameter ‘write-single-snp’ was invoked to reduce linkage among loci by retaining only one SNP per stack for downstream analysis.

Suppl. Table 1. Shortfin mako shark (*Isurus oxyrinchus*) sample and DNA sequencing (mitogenome and SNP) metadata.

Data includes: Sample ID; GenBank Accession Number; Subpop., *a priori* defined subpopulation; Size (FL: Fork Length; cm, centimeters); Sex (F: Female; M: Male); mtDNA: #RR (number of raw Illumina barcoded reads recovered for the sample/individual); mtDNA: #AR (number of Illumina reads contained in reference-based mitogenome assembly for the sample/individual); mtDNA: Length (bp), length in base pairs of complete mitogenome assembly; mtDNA Sequence Depth (minimum; maximum; mean); SNP: #RR, Number of recovered barcoded reads for sample using Stacks; SNP: #PR, Number of recovered barcoded reads for sample following the Stacks module process_radtags; Sample in Final SNP dataset (Yes/No), indicates whether sample is included in final (post-filtered) SNP dataset.

NA, indicates data not available;
-- indicates sample not sequenced;
* indicates pooled number of recovered raw reads across separate Illumina MiSeq runs;
^X^ indicates partial sequence generated via sanger sequencing to fill low coverage mitogenome sequences.

Location abbreviations: WNA, western North Atlantic; GoMex, Gulf of Mexico and Isla Mujeres, Mexico; WSA, western South Atlantic; ENA, eastern North Atlantic; ECA, eastern Central Atlantic; ESA, eastern South Atlantic.

| Sample ID | GenBank Accession Number | Subpop. | Size (FL  cm) | Sex | mtDNA: #RR | mtDNA: #AR | mtDNA: Length (bp) | mtDNA:  Sequence Depth (min; max; mean) | SNP: #RR | SNP: #PR | Sample in Final SNP dataset and proportion of loci typed |
| --- | --- | --- | --- | --- | --- | --- | --- | --- | --- | --- | --- |
| OC-069 | PQ249310 | WSA | NA | F | 301,662 | 187,577 | 16,700 | 138; 5087; 2078 | 8,404,657 | 8,162,387 | No |
| OC-070 | PQ249311 | WSA | NA | M | 284,778 | 168,586 | 16,701 | 54; 8725; 1886 | 17,128,821 | 16,805,326 | No |
| OC-071 | PQ249312 | WSA | NA | F | *1,423,104 | *993,495 | 16,701 | 57; 46588; 11838 | 15,132,476 | 12,092,231 | No |
| OC-072 | PQ249313 | WSA | NA | F | 183,364 | 73,386 | 16,701 | ^X^NA | 13,051,108 | 12,792,497 | Yes (0.739) |
| OC-073 | PQ249314 | WSA | NA | F | 259,246 | 127,154 | 16,701 | 185; 2812; 1380 | 3,591,909 | 3,407,601 | No |
| OC-074 | -- | WSA | NA | F | -- | -- | -- | -- | 7,902,858 | 7,562,760 | Yes (0.900) |
| OC-075 | PQ249315 | WSA | NA | F | 265,156 | 157,526 | 16,700 | 114; 7709; 1705 | 5,349,753 | 5,152,258 | No |
| OC-076 | -- | WSA | NA | F | -- | -- | -- | -- | 7,676,962 | 7,247,010 | Yes (0.803) |
| OC-077 | PQ249316 | WSA | NA | M | 293,760 | 143,964 | 16,699 | 50; 9356; 1623 | 9,623,089 | 9,275,508 | Yes (0.741) |
| OC-078 | -- | WSA | NA | M | -- | -- | -- | -- | 8,158,150 | 8,053,959 | No |
| OC-079 | PQ249317 | WSA | NA | F | 252,160 | 128,033 | 16,699 | 144; 3636; 1324 | R: 5,379,957 R1: 4,380,546 R3: 16,464,265 | R: 5,324,487 R1: 4,321,516 R3: 12,665,877 | Yes (R3: 0.991) |
| OC-080 | PQ249318 | WSA | NA | M | 203,684 | 92,637 | 16,699 | 43; 1967; 992 | 7,379,436 | 7,303,290 | Yes (0.925) |
|  |  |  |  |  |  |  |  |  |  |  |  |
| OC-081 | PQ249319 | WSA | NA | F | 790,084 | 435,362 | 167,00 | 1082; 10880; 5062 | 8,174,292 | 8,033,933 | Yes (0.901) |
| OC-082 | PQ249320 | WSA | NA | M | 285,238 | 163,273 | 16,699 | 115; 6621; 1789 | 7,993,651 | 7,842,443 | Yes (0.916) |
| OC-083 | PQ249321 | WSA | NA | F | 852,242 | 402,582 | 16,699 | 260; 18813; 4493 | 6,875,048 | 6,793,354 | Yes (0.885) |
| OC-084 | -- | WSA | NA | M | -- | -- | -- | -- | 4,094,256 | 4,029,046 | No |
| OC-085 | PQ249322 | WSA | NA | F | 172,550 | 52,298 | 16,699 | 12; 1592; 580 | 6,541,876 | 6,204,601 | Yes (0.914) |
| OC-086 | PQ249323 | WSA | NA | F | 251,874 | 149,783 | 16,699 | 63; 4035; 1709 | 5,797,765 | 5,560,342 | Yes (0.914) |
| OC-087 | PQ249324 | WSA | NA | F | 244,644 | 82,428 | 16,702 | 158; 2115; 869 | 5,642,507 | 5,409,467 | Yes (0.908) |
| OC-088 | PQ249325 | WSA | NA | F | 241,884 | 117,726 | 16,699 | 20; 15159; 1312 | 4,483,614 | 4,367,295 | No |
| OC-095 | PQ249326 | GoMex | NA | M | 196,704 | 43,482 | 16,699 | 11; 2117; 488 | 7,829,427 | 7,610,248 | Yes (0.988) |
| OC-096 | PQ249327 | GoMex | NA | M | 255,148 | 103,064 | 16,700 | 20; 4860; 1109 | 4,703,857 | 4,597,317 | Yes (0.913) |
| OC-097 | -- | GoMex | NA | M | -- | -- | -- | -- | 4,396,715 | 4,300,529 | Yes (0.929) |
| OC-101 | PQ249328 | WNA | 243 | M | 139,558 | 41,781 | 16,700 | 78; 2199; 492 | 7,239,502 | 7,075,908 | Yes (0.937) |
| OC-102 | PQ249329 | WNA | 186 | F | 211,986 | 57,589 | 16,700 | 23; 3191; 683 | 10,217,433 | 10,004,105 | Yes (0.938) |
| OC-103 | PQ249330 | WNA | 174 | M | *795,786 | 176,495 | 16,700 | 22; 13299; 2088 | 6,527,689 | 6,112,407 | Yes (0.928) |
| OC-104 | PQ249331 | WNA | 177 | F | 160,568 | 26,046 | 16,702 | 24; 1027; 292 | 12,559,592 | 12,228,423 | Yes (0.884) |
| OC-110 | -- | WNA | 176 | F | -- | -- | -- | -- | 5,020,162 | 4,744,178 | Yes (0.901) |
| OC-111 | PQ249332 | WNA | 202 | M | 679,854 | 74,334 | 16,699 | 124; 1949; 780 | 12,768,580 | 12,656,290 | Yes (0.939) |
| OC-119 | PQ249333 | WNA | 202 | F | 284,048 | 28,377 | 16,700 | 26; 1198; 325 | 3,109,143 | 3,055,527 | Yes (0.804) |
| OC-120 | PQ249334 | WNA | 191 | F | 187,114 | 72,969 | 16,700 | 47; 2847; 736 | 14,288,563 | 13,769,317 | Yes (0.847) |
| OC-122 | PQ249335 | WNA | 230 | F | 209,806 | 106,259 | 16,700 | 22; 11171; 1228 | R: 6,571,454  R1: 4,660,773 R3: 14,035,883 | R: 6,431,059  R1: 4,461,314 R3: 10,500,719 | Yes (0.922) |
| OC-123 | PQ249336 | WNA | 207 | M | 336,468 | 52,062 | 16,700 | 23; 2375; 558 | 13,150,436 | 13,004,396 | Yes (0.831) |
| OC-124 | PQ249337 | WNA | 180 | M | *1,262,806 | 130,506 | 16,699 | 16; 5877; 1521 | 3,347,680 | 3,218,905 | Yes (0.867) |
| OC-125 | PQ249338 | WNA | 204 | M | *365,102 | 66,272 | 16,700 | 12; 3445; 782 | -- | -- | -- |
| OC-126 | -- | WNA | 205 | M | -- | -- | -- | -- | 7,642,889 | 7,499,795 | Yes (0.882) |
| OC-127 | -- | WNA | 197 | M | -- | -- | -- | -- | 8,642,694 | 8,389,226 | Yes (0.877) |
| OC-128 | -- | WNA | 210 | M | -- | -- | -- | -- | 12,165,948 | 11,975,524 | Yes (0.825) |
| OC-129 | PQ249339 | WNA | 202 | F | 184,076 | 41,703 | 16,700 | 72; 1630; 456 | 5,182,105 | 5,106,125 | Yes (0.893) |
| OC-130 | PQ249340 | WNA | 214 | M | 203,484 | 70,267 | 16,700 | 12; 2513; 721 | 6,136,849 | 6,059,451 | Yes (0.896) |
| OC-131 | PQ249341 | WNA | 196 | F | 286,156 | 55,512 | 16,700 | 18; 3086; 567 | 8,309,960 | 8,163,820 | Yes (0.870) |
| OC-138 | PQ249342 | GoMex | 188 | M | 551,346 | 188,287 | 16,701 | 268; 5864; 2397 | 6,481,868 | 6,386,661 | Yes (0.852) |
| OC-139 | PQ249343 | GoMex | N/A | NA | 198,390 | 53,299 | 16,700 | 11; 2063; 594 | 1,246,689 | 1,208,113 | No |
| OC-155 | -- | WSA | 185 | F | -- | -- | -- | -- | 311,489 | 233,785 | No |
| OC-190 | -- | ECA | 175 | F | -- | -- | -- | -- | 7,234,070 | 7,127,201 | Yes (0.890) |
| OC-191 | -- | ECA | 171 | F | -- | -- | -- | -- | 3,120,574 | 2,868,604 | Yes (0.897) |
| OC-193 | -- | ECA | 159 | M | -- | -- | -- | -- | 9,917,989 | 9,715,335 | Yes (0.979) |
| OC-197 | -- | ECA | 166 | F | -- | -- | -- | -- | 2,922,521 | 2,742,218 | Yes (0.874) |
| OC-198 | -- | ECA | 148 | M | -- | -- | -- | -- | 2,395,463 | 2,278,503 | Yes (0.882) |
| OC-200 | -- | ECA | 154 | F | -- | -- | -- | -- | 6,090,304 | 5,943,774 | Yes (0.912) |
| OC-201 | -- | ECA | 191 | F | -- | -- | -- | -- | 2,186,982 | 2,090,900 | Yes (0.858) |
| OC-204 | PQ249344 | WSA | 173 | F | 1,249,772 | 94,027 | 16,699 | 34; 97907; 11748 | 4,775,492 | 4,693,197 | Yes (0.809) |
| OC-205 | -- | ECA | 236 | M | -- | -- | -- | -- | 361,334 | 290,165 | No |
| OC-206 | -- | WSA | 167 | F | -- | -- | -- | -- | 11,278,896 | 11,117,330 | Yes (0.823) |
| OC-208 | -- | WSA | 164 | F | -- | -- | -- | -- | 5,779,241 | 3,632,472 | Yes (0.848) |
| OC-209 | -- | ECA | 105 | M | -- | -- | -- | -- | 5,577,701 | 5,478,261 | Yes (0.883) |
| OC-210 | -- | ECA | 197 | F | -- | -- | -- | -- | 2,985,272 | 2,848,286 | Yes (0.886) |
| OC-211 | -- | WSA | 165 | M | -- | -- | -- | -- | 13,812,108 | 13,601,368 | Yes (0.985) |
| OC-213 | -- | WSA | 190 | F | -- | -- | -- | -- | 889,836 | 748,749 | No |
| OC-214 | -- | WSA | 140 | F | -- | -- | -- | -- | 5,449,480 | 5,055,675 | Yes (0.921) |
| OC-215 | -- | ECA | 180 | F | -- | -- | -- | -- | 2,550,980 | 2,406,152 | Yes (0.876) |
| OC-217 | -- | ECA | 170 | F | -- | -- | -- | -- | 8,423,590 | 5,502,887 | Yes (0.973) |
| OC-220 | -- | ESA | 159 | M | -- | -- | -- | -- | 275,835 | 209,009 | No |
| OC-222 | -- | ESA | 178 | F | -- | -- | -- | -- | 334,909 | 264,596 | No |
| OC-226 | -- | ESA | 156 | F | -- | -- | -- | -- | 404,721 | 311,436 | No |
| OC-231 | -- | ESA | 160 | F | -- | -- | -- | -- | 345,332 | 196,851 | No |
| OC-235 | -- | ESA | 173 | F | -- | -- | -- | -- | 3,407,348 | 3,256,648 | Yes (0.932) |
| OC-236 | -- | ESA | 155 | M | -- | -- | -- | -- | 10,009,929 | 9,775,697 | Yes (0.805) |
| OC-237 | -- | ESA | 140 | M | -- | -- | -- | -- | 10,646,407 | 10,377,913 | Yes (0.906) |
| OC-239 | -- | ESA | 139 | M | -- | -- | -- | -- | 5,914,928 | 5,661,186 | Yes (0.838) |
| OC-240 | -- | ESA | N/A | M | -- | -- | -- | -- | 2,805,199 | 2,568,441 | No |
| OC-241 | -- | WSA | 207 | F | -- | -- | -- | -- | 141,772 | 54,474 | No |
| OC-248 | -- | WSA | 180 | M | -- | -- | -- | -- | 690,529 | 514,855 | No |
| OC-249 | -- | WSA | 203 | M | -- | -- | -- | -- | 444,957 | 360,998 | No |
| OC-266 | PQ249345 | WNA | 127 | M | 442,764 | 144,503 | 16,700 | 114; 6038; 1608 | 8,281,391 | 8,039,808 | Yes (0.932) |
| OC-269 | -- | WNA | 193 | M | -- | -- | -- | -- | 633,064 | 499,683 | No |
| OC-270 | PQ249346 | WNA | 193 | F | 1,054,768 | 227,652 | 16,700 | 237; 11758; 2745 | 6,531,079 | 6,118,115 | Yes (0.924) |
| OC-271 | PQ249347 | WNA | 172 | F | 188,322 | 18,424 | 16,701 | 28; 822; 209 | 7,118,135 | 6,905,737 | Yes (0.919) |
| OC-299 |  | Mex | NA | F | -- | -- | -- | -- | R: 5,898,963 R1: 5,318,197 R3: 17,206,401 | R: 5,743,622 R1: 5,264,587 R3: 13,269,819 | Yes (R1: 0.902) |
| OC-313 | PQ249348 | GoMex | NA | M | 199,242 | 92,418 | 16,700 | 123; 3413; 1027 | 6,771,004 | 6,684,721 | Yes (0.927) |
| OC-314 | PQ249349 | GoMex | NA | M | 523,084 | 170,043 | 16,700 | 515; 6505; 2020 | 8,301,900 | 8,204,531 | Yes (0.873) |
| OC-316 | PQ249350 | GoMex | NA | M | 661,016 | 192,071 | 16,700 | 81; 7635; 2171 | 12,555,460 | 12,407,947 | Yes (0.943) |
| OC-317 | PQ249351 | GoMex | NA | F | 547,450 | 341,930 | 16,702 | 382; 8419; 4136 | 11,510,510 | 11,384,870 | Yes (0.939) |
| OC-318 | PQ249352 | GoMex | NA | M | 219,640 | 89,055 | 16,702 | 28; 5765; 1008 | 6,686,110 | 6,489,731 | Yes (0.874) |
| OC-319 | PQ249353 | GoMex | NA | M | 236,050 | 102,277 | 16,700 | 57; 4189; 1113 | 7,121,428 | 6,972,823 | Yes (0.865) |
| OC-320 | PQ249354 | GoMex | NA | M | 617,098 | 118,086 | 16,700 | 58; 3983; 1437 | 7,135,657 | 6,843,484 | Yes (0.872) |
| OC-321 | PQ249355 | GoMex | NA | F | 280,240 | 158,158 | 16,701 | 243; 5323; 1782 | 5,842,562 | 5,554,629 | Yes (0.905) |
| OC-323 | PQ249356 | WNA | 239 | F | 229,722 | 71,875 | 16,700 | 17; 4192; 814 | 16,798,412 | 16,249,240 | Yes (0.980) |
| OC-324 | PQ249357 | WNA | 180 | M | 628,264 | 256,434 | 16,700 | 718; 5030; 2685 | 8,685,844 | 8,495,960 | Yes (0.912) |
| OC-325 | PQ249358 | WNA | 198 | F | 661,050 | 243,618 | 16,700 | 332; 11205; 2722 | 11,514,081 | 11,336,481 | Yes (0.925) |
| OC-326 | PQ249359 | WNA | 213 | F | 184,398 | 51,183 | 16,700 | 53; 2516; 577 | 11,178,021 | 10,986,360 | Yes (0.928) |
| OC-327 | -- | WNA | 183 | F | -- | -- | -- | -- | 1,860,517 | 1,653,960 | No |
| OC-328 | PQ249360 | WNA | 153 | M | 186,196 | 71,362 | 16,702 | 79; 2326; 785 | 7,595,224 | 7522,814 | Yes (0.901) |
| OC-347 | PQ249361 | WNA | NA | F | 645,428 | 368,793 | 16,700 | 1231; 9017; 4519 | 12,059,862 | 11749,037 | Yes (0.945) |
| OC-348 | PQ249362 | WNA | NA | M | 575,178 | 255,786 | 16,700 | 376; 5986; 3153 | 18,725,590 | 18,433,549 | Yes (0.980) |
| OC-350 | PQ249363 | GoMex | NA | F | 578,614 | 193,842 | 16,702 | 150; 6320; 2376 | 7594992 | 7519,436 | Yes (0.861) |
| OC-351 | PQ249364 | GoMex | NA | M | 530,756 | 298,909 | 16,699 | 162; 18086; 4203 | 4,873,332 | 4802,804 | Yes (0.907) |
| OC-353 | -- | NA | NA | F | -- | -- | -- | -- | R: 3,519,685 R1: 7,700,378 R3: 18,099,597 | R: 3405,853 R1: 7,341,464 R3: 13,371,665 | No |
| OC-360 | PQ249365 | GoMex | 178 | M | 246,576 | 92,503 | 16,700 | 27; 5136; 1072 | 10,631,530 | 10,379,688 | Yes (0.911) |
| OC-361 | PQ249366 | GoMex | 188 | F | 279,950 | 81,632 | 16,700 | 79; 3527; 886 | 4,116,844 | 4,021,142 | Yes (0.923) |
| OC-362 | PQ249367 | GoMex | 193 | M | 635,424 | 203,868 | 16,699 | 102; 7944; 2500 | 6,516,490 | 6425,736 | Yes (0.875) |
| OC-363 | PQ249368 | GoMex | 146 | F | 294,858 | 116,363 | 16,702 | 110; 6916; 1353 | 7,868,601 | 7,744,043 | Yes (0.924) |
| OC-364 | PQ249369 | GoMex | 177 | F | 225,902 | 76,308 | 16,699 | 22; 3973; 832 | 13,624,433 | 13,325,917 | Yes (0.984) |
| OC-365 | PQ249370 | GoMex | 175 | M | 280,536 | 98,681 | 16,700 | ^X^NA | 10,160,641 | 9,982,399 | Yes (0.919) |
| OC-366 | MZ923832 | ENA | 170 | M | 235,698 | 107,588 | 16,702 | 62; 3070; 1202 | 10,360,969 | 10244,143 | Yes (0.941) |
| OC-367 | PQ249371 | ENA | 215 | F | 249,602 | 145,497 | 16,701 | 198; 4985; 1670 | R: 8,982,373 R1: 4,658,452 | R: 8799,291 R1: 4,552,307 | Yes (0.874) |
| OC-368 | -- | ENA | 175 | M | -- | -- | -- | -- | 3,316,820 | 3244,359 | No |
| OC-369 | PQ249372 | ENA | 160 | M | 154,568 | 72,113 | 16,700 | 91; 1998; 723 | 9,327,774 | 9215,293 | Yes (0.865) |
| OC-370 | PQ249373 | ENA | 170 | M | 247,166 | 200,071 | 16,700 | 146; 10606; 2457 | 11,486,161 | 11192,234 | Yes (0.915) |
| OC-371 | PQ249374 | ENA | 165 | F | 266,306 | 175,545 | 16,700 | 370; 4566; 1972 | 13,202,754 | 13070,548 | Yes (0.914) |
| OC-372 |  | ENA | 160 | M | -- | -- | -- | -- | 3,517,191 | 1,764,376 | No |
| OC-373 |  | ENA | 155 | F | -- | -- | -- | -- | 16,667,703 | 16,178,689 | Yes (0.849) |
| OC-374 |  | ENA | 175 | M | -- | -- | -- | -- | 11,549,494 | 10,979,899 | Yes (0.858) |
| OC-375 | PQ249375 | ENA | 155 | M | 1,124,526 | 628,606 | 16,701 | 12; 29226; 6982 | 5,677,505 | 5,246,802 | Yes (0.899) |
| OC-376 | PQ249376 | ENA | 170 | M | 938,714 | 492,396 | 16,701 | 14; 21591; 5321 | 11,248,046 | 11,047,567 | Yes (0.857) |
| OC-377 |  | ENA | 170 | M | -- | -- | -- | -- | 6,370,767 | 6,190,883 | No |
| OC-378 |  | ENA | 130 | M | -- | -- | -- | -- | 11,893,601 | 11,611,305 | No |
| OC-379 | PQ249377 | ENA | 250 | F | 219,746 | 159,253 | 16,702 | 418; 6065; 1900 | 16,995,928 | 16,614,142 | Yes (0.739) |
| OC-380 |  | ENA | 225 | M | -- | -- | -- | -- | 4,006,940 | 3,857,308 | Yes (0.911) |
| OC-381 | PQ249378 | ENA | 235 | F | 226,886 | 148,650 | 16,700 | 214; 6639; 1807 | 4,022,410 | 3,935,892 | Yes (0.913) |
| OC-382 |  | ENA | 275 | F | -- | -- | -- | -- | 19,101,159 | 18,522,182 | Yes (0.830) |
| OC-383 |  | ENA | 185 | M | -- | -- | -- | -- | 5,974,734 | 5,686,632 | No |
| OC-384 |  | ENA | 240 | F | -- | -- | -- | -- | 5,150,585 | 4,886,511 | No |
| OC-385 |  | ENA | 230 | F | -- | -- | -- | -- | 2,218,922 | 1,669,720 | No |
| OC-386 | PQ249379 | ENA | 315 | F | 293,212 | 125,299 | 16,700 | 55; 4074; 1392 | 16,777,331 | 16,544,728 | Yes (0.773) |
| OC-387 | PQ249380 | ENA | 285 | F | 192,094 | 79,516 | 16,700 | 41; 4297; 887 | 6,882,893 | 6,798,267 | Yes (0.893) |
| OC-388 | -- | ENA | 270 | F | -- | -- | -- | -- | 5,409,497 | 5,255,991 | Yes (0.903) |
| OC-389 | -- | ENA | 270 | F | -- | -- | -- | -- | 7,632,400 | 7,401,254 | Yes (0.824) |
| OC-390 | -- | ENA | 300 | F | -- | -- | -- | -- | 2,344,492 | 2,228,950 | Yes (0.881) |
| OC-397 | PQ249381 | WNA | NA | M | 613,274 | 232,539 | 16,700 | 355; 6270; 2962 | 13,953,872 | 13,726,484 | Yes (0.905) |
| OC-399 | PQ249382 | GoMex | NA | F | 186,958 | 41,092 | 16,700 | 21; 1294; 414 | -- | -- | -- |
| OC-400 | PQ249383 | GoMex | NA | M | 521,528 | 106,187 | 16,700 | 239; 5486; 2073 | -- | -- | -- |
| OC-401 | PQ249384 | GoMex | NA | M | 307,322 | 118,923 | 16,699 | 98; 4154; 1308 | -- | -- | -- |
| OC-402 | PQ249385 | GoMex | NA | M | 214,030 | 97,283 | 16,699 | 152; 2919; 1029 | -- | -- | -- |
| OC-403 | PQ249386 | GoMex | 170 | F | 249,096 | 85,367 | 16,700 | 84; 3789; 962 | 11,327,991 | 11,167,603 | Yes (0.911) |
| OC-404 | PQ249387 | GoMex | 173 | M | 233,194 | 102,097 | 16,699 | 23; 4648; 1167 | 9,944,497 | 9,594,675 | Yes (0.930) |
| OC-405 | PQ249388 | GoMex | 175 | F | 621,422 | 149,585 | 16,700 | 292; 6402; 1615 | 13,212,225 | 12,929,841 | Yes (0.928) |
| OC-406 | PQ249389 | GoMex | 201 | M | 721,742 | 446,776 | 16,700 | 525; 26042; 5767 | 10,617,113 | 10,126,816 | Yes (0.937) |
| OC-407 | PQ249390 | GoMex | 170 | F | 641,794 | 339,272 | 16,700 | 87; 29852; 4649 | 13,238,001 | 13,113,614 | Yes (0.991) |
| OC-408 | -- | GoMex | 170 | F | -- | -- | -- | -- | 9,414,146 | 9,296,928 | Yes (0.936) |
| OC-410 | PQ249391 | WNA | 158 | F | 201,526 | 63,343 | 16,701 | 12; 4241; 641 | -- | -- | -- |
| OC-411 | PQ249392 | WNA | 117 | M | 232,076 | 108,101 | 16,700 | 87; 3757; 1162 | -- | -- | -- |
| OC-413 | PQ249393 | GoMex | NA | NA | 220,610 | 123,813 | 16,700 | 49; 6661; 1310 | -- | -- | -- |
| OC-414 | PQ249394 | GoMex | NA | M | 236,206 | 109,279 | 16,699 | 126; 3883; 1218 | -- | -- | -- |

Suppl. Table 2. Shortfin mako shark mitogenome amplification primers and associated information.

| Fragment | Primer Name | Primer Sequence (5’-3’) | Nucleotide Position (5’-3’) | Length (bp) | Citation Source |
| --- | --- | --- | --- | --- | --- |
| 1 | F: CRF6 R: 16sbrh | F: 5’-CCTAAAGCGTCGACCTTGTA-3’ R: 5’-AACTCAGATCACGTAGGACTAT-3’ | F: 15511-15530 R: 2584-2605 | 3796 | F: Clarke et al. 2015 R: Palumbi et al. 2002 |
| 1A | F: CRF6 R: DASR2 | F: 5’-CCTAAAGCGTCGACCTTGTA-3’ R: 5’-TGCTGAAACTTGCATGTATA-3’ | F: 15511-15530 R: 113-132 | 1323 | F: Clarke et al. 2015 R: Clarke et al. 2015 |
| 1B | F: Mako_16637F R: 16sbrh | F: 5’-CCTAATATACACGGACTCCTCGAAA-3’ R: 5’-AACTCAGATCACGTAGGACTAT-3’ | F: 16596-16620 R: 2584-2605 | 2711 | F: This study R: Palumbi et al. 2002 |
| 2 | F: 16sarl R: Fish_CO1_R1 | F: 5’-AACTCCGCCTGTTTACCAAA-3’ R: 5’-TCTGGATGGCCAAAAAATCAGAACA-3’ | F: 1990-2009 R: 6161-6185 | 4196 | F: Palumbi et al. 2002 R: Ward et al. 2005 |
| 2A | F: 16sarl R: Mako_4611R | F: 5’-AACTCCGCCTGTTTACCAAA-3’  R: 5’-AAATTATTCAACCAAGGTGAGCGAT-3’ | F: 1990-2009 R: 4578-4602 | 2613 | F: Palumbi et al. 2002 R: This study |
| 2B | F: ND2-442-IF Mod R: Fish_CO1_R1 | F: 5’-CCAGGCTCTGCCACACT-3’ R: 5’-TCTGGATGGCCAAAAAATCAGAACA-3’ | F: 4302-4318 R: 6161-6185 | 1884 | F: Naylor et al. 2005* R: Ward et al. 2005 |
| 3 | F: Fish_COI_F1 R: Mako_11401R | F: 5’-TCAACCAACCACAAAGACATTGGCAC-3’ R: 5’-GCCAGAAGTATTGTTCGGCTATG-3’ | F: 5485- 5510 R: 11357-11379 | 5895 | F: Ward et al. 2005 R: This study |
| 3A | F: Fish_COI_F1  R: Mako_8078R | F: 5’-TCAACCAACCACAAAGACATTGGCAC-3’  R: 5’-TGCTGGTTTAGATTTTTCTGCACTT-3’ | F: 5485- 5510 R: 8054-8078 | 2594 | F: Ward et al. 2005 R: This study |
| 3B | F: ATP8.2_L8331_Mod  R: Mako_11401R | R: 5’- AGCGTTAGCCTTTTAAGC-3’ R: 5’-GCCAGAAGTATTGTTCGGCTATG-3’ | F: 7883-7900  R: 11357-11379 | 3497 | F: Sivasundar et al. 2001* R: This study |
| 3B1 | F: ATP8.2_L8331_Mod R: Mako_3B1R | F: 5’-AGCGTTAGCCTTTTAAGC-3’ R: 5’-TCATGGGCTGGGGTCAACTA-3’ | F: 7883-7900 R: 8804-8823 | 941 | F: Sivasundar et al. 2001* R: This study |
| 3B2 | F: Mako_3B2F R: Mako_3BR | F: 5’-ACTATCATACCCGCCGTAGC-3’ R: 5’-CGTAGCCGCAACAGAAGACA-3’ | F: 8642-8661 R: 9647-9666 | 1025 | F: This study R: This study |
| 3B3 | F: Mako_3B3F R: Mako_3B3R | F: 5’-TGAGGCTCATAACTGCTTTTCT-3’ R: 5’-AGAAGGCTGTAGGAGGTGGT-3’ | F: 9550-9571 R: 10421-10440 | 891 | F: This study R: This study |
| 3B4 | F: Mako_3B4F R: Mako_11401R | F: 5’-TTCTAGTAGCCACCTCCCGC-3’ R: 5’-GCCAGAAGTATTGTTCGGCTATG-3’ | F: 10282-10301 R: 11357-11379 | 1098 | F: This study  R: This study |
| 4 | F: Mako_11178F R: Mako_16161R | F: 5’-ATCTGCCTGCGACAAACTGA-3’ R: 5’- AGTTCCCTTTAATGGCACCT-3’ | F: 11168-11187 R: 16130-16149 | 4982 | F: This study R: This study |
| 4A | F: Mako_11178F  R: Mako_13246R | F: 5’-ATCTGCCTGCGACAAACTGA-3’  R: 5’-AAATTTTCCCCGATTCAACCCAC-3’ | F: 11168-11187  R: 13236-13258 | 2091 | F: This study R: This study |
| 4A1 | F: Mako_4A1F R: Mako_4A1R | F: 5’-GGTCTCGCACTCAACCGTTA-3’  R: 5’-CAATTGGGGCTTCAACGTGG-3’ | F: 10104-10123 R: 11102-11021 | 918 | F: This study R: This study |
| 4A2 | F: Mako_4A2F R: Mako_4A2R | F: 5’-CTCGGCACCCTGTCCATAAT-3’ R: 5’-ACAGAGTGTCCAGCCTCAGA-3’ | F: 10856-10875 R: 11706-11725 | 870 | F: This study R: This study |
| 4A3 | F: Mako_4A3F  R: Mako_4A3R | F: 5’-ATCTCCTCCTTAGCCTCCACC-3’ R: 5’-CGAGGAGGGGGAAGGTTAGA-3’ | F: 11646-11666 R: 12564-12583 | 938 | F: This study R: This study |
| 4A4 | F: Mako_4A4F R: Mako_4A4R | F: 5’-CGAGTAGGGGATATCGGACT-3’ R: 5’-TAGTCACCAGTAGGGCGGAG-3’ | F: 12463-12482 R: 13404-13423 | 961 | F: This study R: This study |
| 4A5 | F: Mako_4A5F R: Mako_4A5R | F: 5’- TCAACCCGCTCTCCCCTATT-3’ R: 5’-GGGGTAGGGTGCGGTTTATT-3’ | F: 13250-13269 R: 14151-14170 | 921 | F: This study R: This study |
| 4B | F: Mako_12977F R: Mako_16161R | F: 5’-TCTGGGTCTATCATCCACAGTCT-3’  R: 5’- AGTTCCCTTTAATGGCACCT-3’ | F: 12967-12989 R: 16130-16149 | 3183 | F: This study R: This study |
| 4B1 | F: Mako_12977F R: Mako_4A5R | F: 5’-TCTGGGTCTATCATCCACAGTCT-3’ R: 5’-GGGGTAGGGTGCGGTTTATT-3’ | F: 12967-12989 R: 14151-14170 | 1204 | F: This study R: This study |
| 4B2 | F: Mako_4B2F R: Mako_4B2R | F: 5’-TCCTCCACCCCTGATCAACT-3’ F: 5’-TCAAGGTAGGACGTAGCCCA-3’ | F: 13923-13942 R: 14739-14758 | 836 | F: This study R: This study |
| 4B3 | F: Mako_4B3F R: Mako_4B3R | F: 5’-TCTACTTTCACATCGCCCGAG-3’ R: 5’-AAGGGGCAATGCAATGAGGA-3’ | F: 14634-14654 R: 15438-15457 | 824 | F: This study R: This study |
| 4B4 | F: Mako_4B4F R: Mako_1616R | F: 5’-GCACCTTTCGCCCACTTACA-3’ R: 5’-AGTTCCCTTTAATGGCACCT-3’ | F: 15297-15316 R: 16130-16149 | 853 | F: This study R: This study |

Abbreviations: Fragment (Fragment Name or Number); Primer Name (F: Forward, R: Reverse); Nucleotide Position (5’-3’) [5’ and 3’ end nucleotide position of primer sequence with respect to shortfin mako reference sequence: KF361861 (Chang et al. 2015)]; Length (bp) (Length of amplified fragment in base pairs with respect to KF361861; Citation Source (Reference of Primer Sequence if previously published, * = modified herein from original sequence).

Suppl. Table 3a. Shortfin mako sharks Analysis of Molecular Variance (AMOVA) for 86 complete mitogenome sequences, with subpopulations grouped hierarchically (and latitudinally) according to current ICCAT stock boundary (5°N). Group 1: Northern Atlantic sharks (WNA, GoMex, ENA), and Group 2: Southern Atlantic sharks (WSA); analysis was performed assuming pairwise genetic distance (*ϕ*) and with 10 000 permutations to determine significance of variance components.

| Sources of Variation | DF | Sum Sq. | % variance | *ϕ*-statistic | *P*-value |
| --- | --- | --- | --- | --- | --- |
| Between Hierarchical Groups  (Northern Atlantic vs. Southern Atlantic) | 1 | 226.168 | 8.43 | 0.084 | 0.248 |
| Between Subpops Within Hierarchical Groups  [Northern (WNA, GoMex, ENA); Southern (WSA)] | 2 | 194.139 | 3.65 | 0.040 | 0.067 |
| Within Subpops | 82 | 4219.426 | 87.93 | 0.121 | **0.008** |
| Total | 85 | 4639.733 |  |  |  |

Abbreviations: DF, degrees of freedom; Sum Sq., sum of squares; % variance, percent variance.
Bold values indicate significance at *P*<0.05

Suppl. Table 3b. Shortfin mako sharks Analysis of Molecular Variance (AMOVA) for 86 complete mitogenome sequences, with subpopulations grouped hierarchically (and longitudinally) according to ICCAT stock/statistical area boundaries for sharks (40°W as stock boundary in North; 20-30°W as stock boundary in South). Group 1: Western Atlantic sharks (WNA, GoMex, WSA), and Group 2: Eastern Atlantic sharks (ENA); analysis was performed assuming pairwise genetic distance (*ϕ*) and with 10 000 permutations to determine significance of variance components.

| Sources of Variation | DF | Sum Sq. | % variance | *ϕ*-statistic | *P*-value |
| --- | --- | --- | --- | --- | --- |
| Between Hierarchical Groups  (Western Atlantic vs. Eastern Atlantic) | 1 | 88.025 | -2.34 | -0.023 | 0.754 |
| Between Subpops Within Hierarchical Groups  [Western (WNA, GoMex, WSA); Eastern (ENA)] | 2 | 332.282 | 8.60 | 0.084 | **0.009** |
| Within Subpops | 82 | 4219.426 | 93.74 | 0.063 | **0.008** |
| Total | 85 | 4639.733 |  |  |  |

Abbreviations: DF, degrees of freedom; Sum Sq., sum of squares; % variance, percent variance.
Bold values indicate significance at *P*<0.05

Suppl. Table 4. Total number and percentage of remaining Single Nucleotide Polymorphisms (SNPs) and individual shortfin mako shark samples after sequential data quality filtering steps outlined in the Materials and Methods section.

| Filtering Step | Number of SNPs | % of remaining SNPs | Number of individuals |
| --- | --- | --- | --- |
| No filter | 168 339 | 100% | 144 |
| Loci with <8X coverage, set to 0 | 168 339 | 100% | 144 |
| Loci > 30% missing data and MAF <0.05 | 5302 | 3.15 | 144 |
| Individuals > 30% missing data | 5302 | 3.15 | 115 |
| Loci with mean depth across individuals < 10X | 5302 | 3.15 | 115 |
| Loci > 30% missing data and MAF <0.05 | 5248 | 3.12 | 115 |
| Loci with a max mean depth of > 2X mean coverage across loci | 5202 | 3.09 | 115 |
| Removal of sample duplicates (technical replicates; *n* = 9) and sample with missing capture coordinates (*n* = 1) | 5202 | 3.09 | 105 |

Abbreviation: MAF, minor allele frequency.

Suppl. Table 5. Summary of single- and compound-outlier clusters (SOCs, and COCs, respectively) identified by LDna within the shortfin mako sharks SNP dataset.

| Type of Cluster | Name of Cluster | n_loci_ | \|E\| | λ | Median LD (MAD) | Hypothesized origin |
| --- | --- | --- | --- | --- | --- | --- |
| SOC | 500_0.23 | 230 | 9635 | 18.40 | 0.148 (0.118) | Inversion |
| COC | 609_0.22 | 282 | 10063 | 8.46 | 0.063 (0.060) | -- |

Abbreviations: n_loci_, number of loci contained in cluster; |E|, number of edges or connections between loci contained within cluster; λ, the change in median LD between all loci in a cluster before and after merger; Median LD, median of pairwise LD values within the outlier cluster; MAD, unscaled median absolute deviation of the intra-cluster pairwise LD values.

Suppl. Table 6. Mitogenome genetic diversity estimates and conservation status in elasmobranchs.

| Species | Surveyed Range | n | *h* | π | Global IUCN Conservation Status | Citation |
| --- | --- | --- | --- | --- | --- | --- |
| Shortfin mako (*Isurus oxyrhinchus*) | Atlantic-wide | 86 | 0.997 ± 0.002 | 0.00654 ± 0.00314 | Endangered | This study |
| Scalloped hammerhead  (*Sphyrna lewini*) | Mexican Pacific | 191 | 0.929 ± 0.012 | 0.00021 ± 0.00012 | Critically Endangered | Rangel-Morales et al. 2022 |
| Speartooth shark  (*Glyphis glyphis*) | Northern Australia river drainages | 93 | 0.76 | 0.00019 | Vulnerable | Feutry et al. 2014 |
| Largetooth sawfish  (*Pristis pristis*) | Northern Australia river drainages | 92 | 0.919 | 0.0011 | Critically Endangered | Feutry et al. 2015 |
| Great hammerhead (*Sphyrna mokarran*) | Global | 169 | 0.993 ± 0.002 | 0.00274 ± 0.00132 | Critically Endangered | Bernard and Shivji (unpublished data) |
| Angelshark (*Squatina squatina*) | Canary Islands and coastal waters of Ireland, UK | 9 | 1.00 ± 0.052 | 0.000879 ± 0.000494 | Critically Endangered | Fitzpatrick 2018 |
| Basking shark  (*Cetorhinus maximus*) | Global | 34 | 0.970 ± 0.014 | 0.0014 ± 0.000 | Endangered | Finnegan 2014 |
| Bull shark (*Carcharhinus plumbeus*) | W. Atlantic | 51 | 0.730 – 1.000 | 0.00005 – 0.0004 | Vulnerable | Laurrabaquio-Alvarado et al. 2021 |
| Bull shark (*Carcharhinus plumbeus*) | Indo-Pacific | 361 | 0.890 | 0.001 | Vulnerable | Devloo-Delva et al. 2023 |
| Thorny skate (*Amblyraja radiata*) | North Atlantic | 527 | 0.9992 ± 0.0002 | 0.0063 ± 0.0030 | Vulnerable | Denton et al. 2024 |


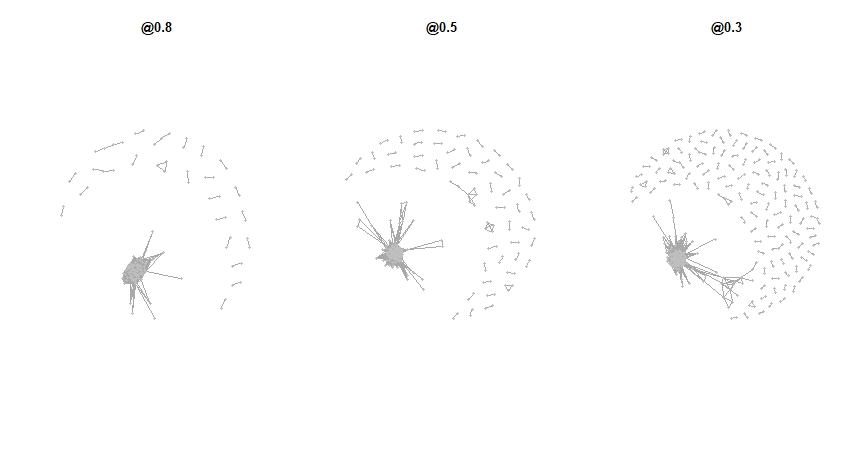

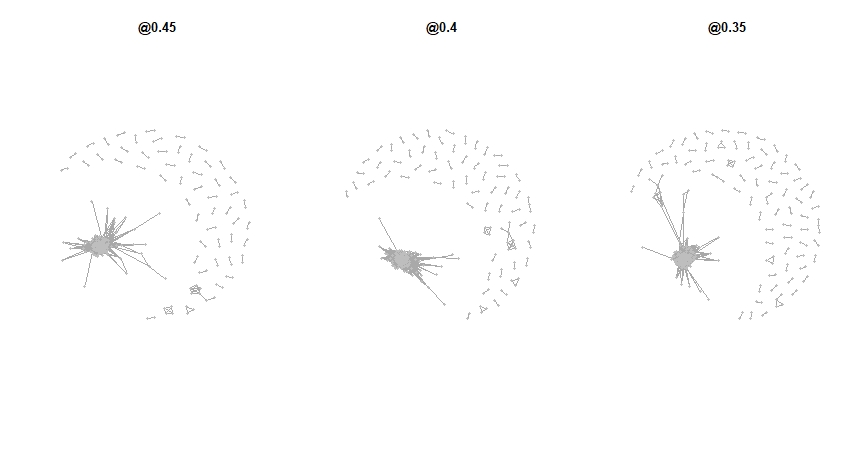


Suppl. Fig. 1. Visualization in R of the linkage disequilibrium network analysis (LDna 0.64; Kemppainen et al. 2015) of the shortfin mako sharks RAD dataset depicting the full network (function: ‘plotLDnetwork’) at six individual linkage disequilibrium (LD) thresholds (*_r_*_2_): 0.8, 0.5, 0.3, 0.45, 0.4, 0.35.

**Literature Cited**

Chang CH, Shao K-T, Lin Y-S, Tsai A-Y, Su P-X, Ho H-C. (2015) The complete mitochondrial genome of the shortfin mako, Isurus oxyrinchus (Chondrichthyes, Lamnidae). Mitochondrial DNA, 26(3): 475–476. <https://doi.org/10.3109/19401736.2013.834430>

Clarke CR, Karl SA, Horn RL, Bernard AM, Lea JS, Hazin FH, Prodöhl, Shivji MS. (2015) Global mitochondrial DNA phylogeoraphy and population structure of the silky shark, *Carcharhinus falciformis*. Marine Biology, 162: 945-955. <https://doi.org/10.1007/s00227-015-2636-6>

Denton JSS, Kneebone J, Yang L, Lynghammar A, McElroy D, Corrigan S, Jakobsdóttir K, Miri C, Simpson M, Naylor GJP. (2004) Mitogenomic evidence of population differentiation of thorny skate, Amblyraja radiata, in the North Atlantic. Journal of Fish Biology, 104(5): 1513-1524. <https://doi.org/10.1111/jfb.15689>

[Devloo-Delva](https://onlinelibrary.wiley.com/authored-by/Devloo%E2%80%90Delva/Floriaan) F, [Burridge](https://onlinelibrary.wiley.com/authored-by/Burridge/Christopher+P.) CP, [Kyne](https://onlinelibrary.wiley.com/authored-by/Kyne/Peter+M.) PM, [Brunnschweiler](https://onlinelibrary.wiley.com/authored-by/Brunnschweiler/Juerg+M.) JM, [Chapman](https://onlinelibrary.wiley.com/authored-by/Chapman/Demian+D.) DD, [Charvet](https://onlinelibrary.wiley.com/authored-by/Charvet/Patricia) P, [Chen](https://onlinelibrary.wiley.com/authored-by/Chen/Xiao) X, [Cliff](https://onlinelibrary.wiley.com/authored-by/Cliff/Geremy) G, [Daly](https://onlinelibrary.wiley.com/authored-by/Daly/Ryan) R, [Drymon](https://onlinelibrary.wiley.com/authored-by/Drymon/J.+Marcus) JM, [Espinoza](https://onlinelibrary.wiley.com/authored-by/Espinoza/Mario) M, [Fernando](https://onlinelibrary.wiley.com/authored-by/Fernando/Daniel) D, [Garcia Barcia](https://onlinelibrary.wiley.com/authored-by/Barcia/Laura+Garcia) L, [Glaus](https://onlinelibrary.wiley.com/authored-by/Glaus/Kerstin) K,  [González-Garza](https://onlinelibrary.wiley.com/authored-by/Gonz%C3%A1lez%E2%80%90Garza/Blanca+I.) BI, [Grant](https://onlinelibrary.wiley.com/authored-by/Grant/Michael+I.) MI, [Gunasekera](https://onlinelibrary.wiley.com/authored-by/Gunasekera/Rasanthi+M.) RM, [Hernandez](https://onlinelibrary.wiley.com/authored-by/Hernandez/Sebastian) S, [Hyodo](https://onlinelibrary.wiley.com/authored-by/Hyodo/Susumu) S, [Jabado](https://onlinelibrary.wiley.com/authored-by/Jabado/Rima+W.) RW, [Jaquemet](https://onlinelibrary.wiley.com/authored-by/Jaquemet/S%C3%A9bastien) S, [Johnson](https://onlinelibrary.wiley.com/authored-by/Johnson/Grant) G, [Ketchum](https://onlinelibrary.wiley.com/authored-by/Ketchum/James+T.) JT, [Magalon](https://onlinelibrary.wiley.com/authored-by/Magalon/H%C3%A9l%C3%A8ne) H, [Marthick](https://onlinelibrary.wiley.com/authored-by/Marthick/James+R.) JR, [Mollen](https://onlinelibrary.wiley.com/authored-by/Mollen/Frederik+H.) FH,  [Mona](https://onlinelibrary.wiley.com/authored-by/Mona/Stefano) S, [Naylor](https://onlinelibrary.wiley.com/authored-by/Naylor/Gavin+J.+P.) GJP, [Nevill](https://onlinelibrary.wiley.com/authored-by/Nevill/John+E.+G.) JEG, [Phillips](https://onlinelibrary.wiley.com/authored-by/Phillips/Nicole+M.) NM, [Pillans](https://onlinelibrary.wiley.com/authored-by/Pillans/Richard+D.) RD, [Postaire](https://onlinelibrary.wiley.com/authored-by/Postaire/Bautisse+D.) BD, [Smoothey](https://onlinelibrary.wiley.com/authored-by/Smoothey/Amy+F.) AF, [Tachihara](https://onlinelibrary.wiley.com/authored-by/Tachihara/Katsunori) K, [Tillet](https://onlinelibrary.wiley.com/authored-by/Tillet/Bree+J.) BJ, [Valerio-Vargas](https://onlinelibrary.wiley.com/authored-by/Valerio%E2%80%90Vargas/Jorge+A.) JA, [Feutry](https://onlinelibrary.wiley.com/authored-by/Feutry/Pierre) P. (2023) From rivers to ocean basins: The role of ocean barriers and philopatry in the genetic structuring of a cosmopolitan coastal predator. Ecology and Evolution, 13(2): e9837. <https://doi.org/10.1002/ece3.9837>

Feutry P, Kyne P, Pillans RD, Chen X, Marthick JR, Morgan DL, Grewe PM. (2015) Whole mitogenome sequencing refines population structure of the critically endangered sawfish *Pristis pristis.* Marine Ecology Progress Series, 533: 237-244. <https://doi.org/10.3354/meps11354>

Feutry P, Kyne PM, Pillans RD, Chen X, Naylor GJP, Grewe PM. (2014) Mitogenomics of the speartooth shark challenges ten years of control region sequencing. BMC Evolutionary Biology, 14: 232. <https://doi.org/10.1186/s12862-014-0232-x>

Finnegan KA. (2014) A mitogenomics view of the population structure and evolutionary history of the basking shark *Cetorhinus maximus*. Master’s Thesis. Nova Southeastern University.

Fitzpatrick CK. (2018) Genetic population and evolutionary dynamics of the angel sharks, *Squantina* spp. Master’s Thesis. Nova Southeastern University.

Gorman J, Marra N, Shivji MS, Stanhope MJ. (2019) The complete mitochondrial genome of an Atlantic Ocean shortfin mako shark, *Isurus oxyrinchus.* Mitochondrial DNA Part B, 4: 2, 3642-3643. <https://doi.org/10.1080/23802359.2019.1677524>

Kemppainen P, Knight CG, Sarma DK, Hlaing T, Prakash A, Naung YMM, Somboon P, Mahanta J, Walton C. (2015) Linkage disequilibrium network analysis (LDna) gives a global view of chromosomal inversions, local adaptation and geographic structure. Molecular Ecology Resources, 15: 1031-1045. <https://doi.org/10.1111/1755-0998.12369>

Laurrabaquio-Alvarado NS, Díaz-Jaimes P, Hinojosa-Álvarez S, Blanco-Parra MDP, Adams DH, Pérez-Jiménez JC, Castillo-Géniz JL. (2021) Mitochondrial DNA genome evidence for the existence of a third divergent lineage in the western Atlantic Ocean for the bull shark (*Carcharhinus leucas*). Journal of Fish Biology, 99(1): 275-282. <https://doi.org/10.1111/jfb.14698>

Naylor GJP, Ryburn JA, Fedrigo O, Lopez JA. (2005) Phylogenetic relationships among the major lineages of modern elasmobranchs. Reproductive Biology and Phylogeny, 3: 1- 25.

Paris JR, Stevens JR, Catchen JM. (2017) Lost in parameter space: a road map for STACKS. Methods in Ecology and Evolution, 8(10): 1360-1373. <https://doi.org/10.1111/2041-210X.12775>

Palumbi S, Martin A, Romano S, McMillan WO, Stice L, Grabowski G. (2002). The Simple Fool’s Guide to PCR. Simple Fool’s Guide.

Rangel-Morales JM, Rosales-López LP, Díaz-Jaimes P, Amezcua-Martínez F, Ketchum JT, Hoyos-Padilla M, Corgos A. (2022) Regional philopatry of scalloped hammerhead sharks (Sphyrna lewini) in nursery areas in the Mexican Pacific. Hydrobiologia, 849: 3083-3099. <https://doi.org/10.1007/s10750-022-04880-2>

Rochette NC, Catchen JM. (2017) Deriving genotypes from RAD-seq short-read data using Stacks. Nature Protocols, 12: 2640-2659. <https://doi.org/10.1038/nprot.2017.123>

Sivasundar A, Bermingham E, Ortí G. (2001) Population structure and biogeography of migratory freshwater fishes (Prochilodus: Characiformes) in major South American rivers. Molecular Ecology, 10(2): 407-417. <https://doi.org/10.1046/j.1365-294X.2001.01194.x>

Ward RD, Zemlak TS, Innes BH, Last PR, Herbert PDN. (2005) DNA Barcoding Australia's Fish Species. Philosophical Transactions of the Royal Society B-Biological Sciences, 360(1462): 1847-1857. <https://doi.org/10.1098/rstb.2005.1716>
